# Multiplicity of cerebrospinal fluid functions: New challenges in health and disease

**DOI:** 10.1186/1743-8454-5-10

**Published:** 2008-05-14

**Authors:** Conrad E Johanson, John A Duncan, Petra M Klinge, Thomas Brinker, Edward G Stopa, Gerald D Silverberg

**Affiliations:** 1 Department of Clinical Neurosciences, Warren Alpert Medical School at Brown University, Providence, RI 02903, USA; 2 International Neuroscience Institute Hannover, Rudolph-Pichlmayr-Str. 4, 30625 Hannover, Germany

## Abstract

This review integrates eight aspects of cerebrospinal fluid (CSF) circulatory dynamics: formation rate, pressure, flow, volume, turnover rate, composition, recycling and reabsorption. Novel ways to modulate CSF formation emanate from recent analyses of choroid plexus transcription factors (E2F5), ion transporters (NaHCO_3 _cotransport), transport enzymes (isoforms of carbonic anhydrase), aquaporin 1 regulation, and plasticity of receptors for fluid-regulating neuropeptides. A greater appreciation of CSF pressure (CSFP) is being generated by fresh insights on peptidergic regulatory servomechanisms, the role of dysfunctional ependyma and circumventricular organs in causing congenital hydrocephalus, and the clinical use of algorithms to delineate CSFP waveforms for diagnostic and prognostic utility. Increasing attention focuses on CSF flow: how it impacts cerebral metabolism and hemodynamics, neural stem cell progression in the subventricular zone, and catabolite/peptide clearance from the CNS. The pathophysiological significance of changes in CSF volume is assessed from the respective viewpoints of hemodynamics (choroid plexus blood flow and pulsatility), hydrodynamics (choroidal hypo- and hypersecretion) and neuroendocrine factors (i.e., coordinated regulation by atrial natriuretic peptide, arginine vasopressin and basic fibroblast growth factor). In aging, normal pressure hydrocephalus and Alzheimer's disease, the expanding CSF space reduces the CSF turnover rate, thus compromising the CSF sink action to clear harmful metabolites (e.g., amyloid) from the CNS. Dwindling CSF dynamics greatly harms the interstitial environment of neurons. Accordingly the altered CSF composition in neurodegenerative diseases and senescence, because of adverse effects on neural processes and cognition, needs more effective clinical management. CSF recycling between subarachnoid space, brain and ventricles promotes interstitial fluid (ISF) convection with both trophic and excretory benefits. Finally, CSF reabsorption via multiple pathways (olfactory and spinal arachnoidal bulk flow) is likely complemented by fluid clearance across capillary walls (aquaporin 4) and arachnoid villi when CSFP and fluid retention are markedly elevated. A model is presented that links CSF and ISF homeostasis to coordinated fluxes of water and solutes at both the blood-CSF and blood-brain transport interfaces.

**Outline:**

1 Overview

2 CSF formation

2.1 Transcription factors

2.2 Ion transporters

2.3 Enzymes that modulate transport

2.4 Aquaporins or water channels

2.5 Receptors for neuropeptides

3 CSF pressure

3.1 Servomechanism regulatory hypothesis

3.2 Ontogeny of CSF pressure generation

3.3 Congenital hydrocephalus and periventricular regions

3.4 Brain response to elevated CSF pressure

3.5 Advances in measuring CSF waveforms

4 CSF flow

4.1 CSF flow and brain metabolism

4.2 Flow effects on fetal germinal matrix

4.3 Decreasing CSF flow in aging CNS

4.4 Refinement of non-invasive flow measurements

5 CSF volume

5.1 Hemodynamic factors

5.2 Hydrodynamic factors

5.3 Neuroendocrine factors

6 CSF turnover rate

6.1 Adverse effect of ventriculomegaly

6.2 Attenuated CSF sink action

7 CSF composition

7.1 Kidney-like action of CP-CSF system

7.2 Altered CSF biochemistry in aging and disease

7.3 Importance of clearance transport

7.4 Therapeutic manipulation of composition

8 CSF recycling in relation to ISF dynamics

8.1 CSF exchange with brain interstitium

8.2 Components of ISF movement in brain

8.3 Compromised ISF/CSF dynamics and amyloid retention

9 CSF reabsorption

9.1 Arachnoidal outflow resistance

9.2 Arachnoid villi vs. olfactory drainage routes

9.3 Fluid reabsorption along spinal nerves

9.4 Reabsorption across capillary aquaporin channels

10 Developing translationally effective models for restoring CSF balance

11 Conclusion

## 1 Overview

**Figure 1:**
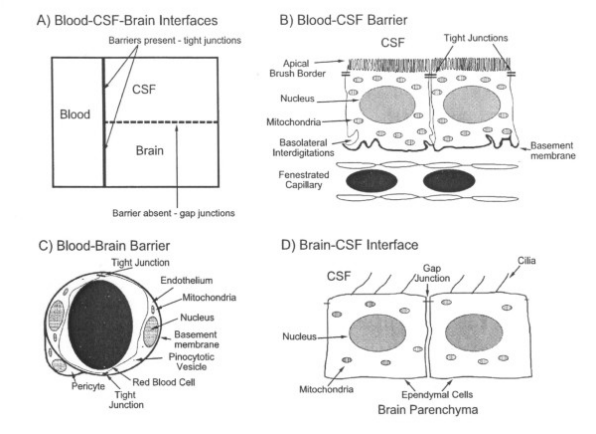
**Morphology of blood-brain-CSF interfaces:** (A) Schema of main CNS compartments and interfaces. The blood-brain and blood-CSF barriers are true barriers with tight junctions between endothelial and epithelial cells, respectively. The brain-CSF interface, because of gap junctions between ependymal (or pia-glial) cells, is more permeable than brain or spinal cord capillaries and choroid plexus. (B) Blood-CSF barrier. CP is comprised of one cell layer of circumferentially arranged epithelial cells. Plexus capillaries, unlike counterparts in brain, are permeable to macromolecules. (C) Blood-brain barrier: Endothelial cells are linked by tight junctions, conferring low paracellular permeability. Endothelial cell pinocytotic vesicle paucity reflects minimal transcytosis. (D) Brain-CSF interface: Ependymal lining in lateral ventricles permits relatively free diffusion of solutes between brain ISF and large-cavity CSF. Motile cilia at ependymal cell apex move CSF downstream to SAS. Reprinted with permission from *Advanced Drug Delivery Reviews *[17].

Free-flowing cerebrospinal fluid (CSF) finely-regulated in composition is vital to brain health [1,2]. Aging- or disease-induced alterations in CSF circulation adversely impact neuronal performance [2,3]. Throughout life the choroid plexus (CP)-CSF dynamics are damaged by tumors, infections, trauma, ischemia or hydrocephalus [4-8]. Severely disrupted CSF flow disturbs cognitive and motor functions [9]. CNS viability is taxed if key choroidal-CSF parameters are distorted. For health and disease it is essential to delineate interactions of CP and brain with the intervening CSF (Fig. 1).

Improved biotechnology and models can now precisely evaluate the role of the CP-CSF in modulating brain development, metabolism and aging [10-12]. Alternative transplant [13-15] and pharmacological strategies [16-18] utilizing the CP may be helpful in rescuing the debilitated CSF system. Such therapy to rectify CSF parameters would ultimately benefit neuronal network functions. This review sets forth new approaches to stabilize the CSF in disease or curb its deterioration in late-life stages [19].

**Figure 2:**
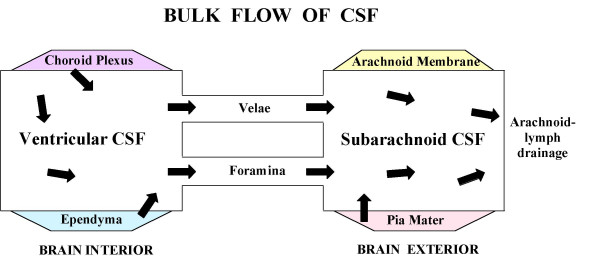
**Large-cavity CSF compartments and bulk flow.** Extracellular fluid enables volume transmission (convection) of fluid from ventricles to SAS [7, 27]. CSF formed by lateral, 3^rd ^and 4^th ^ventricle CPs flows from the lateral to 3^rd ^ventricle *via *the cerebral aqueduct and 4^th ^ventricle to SAS in cisterna magna. Then CSF is transmitted by bulk flow through cisternal foramina (Magendie and Luschka) into basal cisterns. CSF is also convected from ventricles through velae channels to the quadrigeminal and ambient cisterns [167]. Thereafter fluid is convected to the SAS of the spinal cord and brain convexities. As CSF flows through the ventriculo-subarachnoid system, there are diffusional and bulk flow exchanges between CSF and brain [16, 24, 168, 169, 289], depending upon region-specific gradients for concentration and hydrostatic pressure that promote widespread distribution of CSF-borne materials [255]. Normally CSF is readily distributed from the ventricles to arachnoidal drainage sites. In hydrocephalus, flow pathways can be disrupted at multiple points.

CSF assessment should collectively consider physical, biochemical and physiological parameters (Table 1). In addition, the large-cavity CSF system is multi-compartmental (Fig. 2). Ventricles, cisterns and subarachnoid space (SAS) are serially linked. Upstream CSF transport phenomena exert effects downstream. Ventricular CSF is functionally interactive with the ependymal wall [1] as well as the more deeply-lying brain capillary surfaces [16,17]. Diffusion and bulk flow promote solute and water distribution both into and out of brain, depending upon prevailing regional gradients for concentration and hydrostatic pressure in various compartments (Fig. 1). It is thus pertinent to analyze CNS fluid spaces- how they are disrupted by ventricular dysfunction and might be modified by CSF-borne therapeutic agents. This should promote translational steps for managing CSF in neurodegenerative diseases and other disorders.

**Table 1:** Physico-chemical properties and physiological parameters of the CSF system

**CSF Formation Rate**	Produced mainly by choroid plexuses, CSF is formed at 0.4 ml/min/g in several mammals. Human production rates vary from 0.3 to 0.6 ml/min depending upon measurement method. CSF formation, an active secretion by epithelial cells, involves pumps, cotransporters & antiporters, ion channels and aquaporins [1, 77]. It is under neuroendocrine & hormonal modulation [6, 246]. Daily volume of CSF produced in adult humans is 500–600 ml.
**CSF Pressure**	In adult humans the normal CSFP is about 100 mm H_2_O. Ventricular pressure is normally about 35 mm H_2_0 in rats. CSFP is typically slightly higher than venous pressure in the dural sinuses. CSFP is stable when CSF formation and reabsorption are balanced. Elevated CSFP is reduced by acetazolamide, which inhibits formation of fluid by the choroid plexus [36, 43, 44].
**CSF Flow**	Flow of CSF is pulsatile [185, 192]. CSF pulsations depend upon the arterial hemodynamics in the plexus. CSF flow is from the lateral to 3^rd ^and 4^th ^ventricles. CSF flows out of 4^th ^ventricular foramina into basal cisterns [1]. It is then convected into the spinal and cortical subarachnoid spaces.
**CSF Volume**	In healthy humans, the ventricular and subarachnoid CSF spaces, respectively, are about 25% and 75% of total CSF volume [1]. Total CSF space in young adults is about 160 ml, i.e., more than half that of brain interstitial fluid volume. The ratio of CSF volume to brain volume increases in aging and neurodegeneration [9, 19].
**CSF Turnover Rate**	CSF turnover rate is directly proportional to CSF formation rate and inversely related to CSF volume [3]. It is an index of CSF sink action on brain interstitial solutes [33]. Clearance of brain metabolites depends on a CSF renewal of 0.3–0.4% per min. Mammalian CSF is totally replaced about 4 times each day.
**CSF Composition**	CSF is an active secretion, not simply a plasma ultrafiltrate [1]. Carrier transport of ions and molecules, along with molecular sieving at blood-CSF barrier, generates a CSF concentration lower than plasma in protein, K [221] & urea [33]; and higher in Cl & Mg. Disease distorts CSF chemistry, enabling CSF biomarking [227]. CSF is 99% water, compared to the 92% water of plasma.
**CSF Recycling**	In addition to CSF macrocirculation through ventriculo-subarachnoid spaces, there is limited microcirculation of CSF recirculated by bulk flow from the cortical subarachnoid space into Virchow-Robin perivascular spaces and then out of brain via CSF drainage routes [253–255].
**CSF Reabsorption**	CSF is cleared from CNS by bulk flow along sleeves of the subarachnoid space surrounding cranial nerves that enter the nose and eyes [263, 264]. Substantial drainage occurs through the cribriform plate, the CSF eventually reaching the nasal submucosa and downstream cervical lymphatics [267, 268]. CSF is also cleared along spinal nerves [279]. Lymphatic drainage of CSF needs substantiation in humans. Arachnoid villi in dural venous sinuses may serve as ancillary drainage sites when CSFP is elevated.

## 2 CSF formation

Several CNS regions form CSF or a CSF-like fluid. Although CP tissues generate about two thirds of the total production, extrachoroidal sources make up the balance [20]. The capillary-astrocyte complex in the blood-brain barrier (BBB) has been implicated as an active producer of brain interstitial fluid (ISF) [21], but normally at a rate substantially slower (1/100 when normalized for barrier surface area) than the blood-cerebrospinal fluid barrier BCSFB [22]. The working hypothesis that the BBB is a fluid generator, although attractive, needs substantiation. Another likely source of CSF, according to Pollay and Curl, is the ependyma lining the ventricles [23]. The arachnoid membrane with its relatively large surface area, although secreting peptides and proteins [24], probably does not actively form CSF. Because CP rapidly produces most of fluid within the CNS and has been extensively documented for CSF-forming capabilities [1], the discussion below focuses on choroidal epithelium.

**Figure 3:**
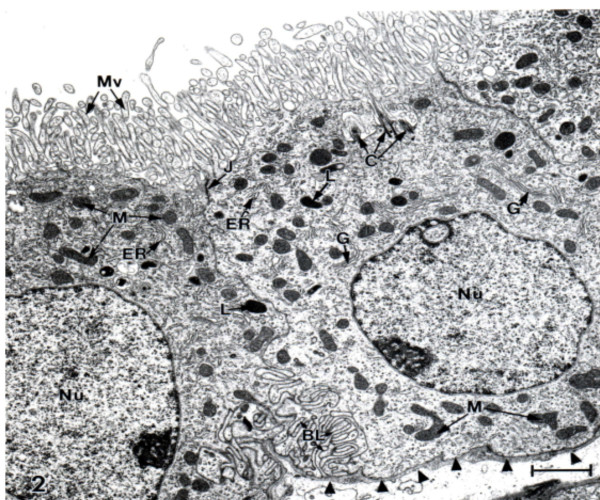
**Ultrastructure of choroid epithelium.** CP from a lateral ventricle of an untreated adult Sprague-Dawley rat was fixed for electron microscopy with OsO4. There is a profusion of apical membrane (CSF-facing) microvilli (Mv) and many intracellular mitochondria (M). J refers to the tight junction welding two cells at their apical poles. C = centriole. G and ER, Golgi apparatus and endoplasmic reticulum. Nucleus (Nu) is oval and has a nucleolus. Arrowheads point to basal lamina at the plasma face of the epithelial cell; the basal lamina separates the choroid cell above from the interstitial fluid below. Basal labyrinth (BL) is the intertwining of basolateral membranes of adjacent cells. Choroidal morphology resembles proximal tubule, consistent with both cell types rapidly turning over fluid. Scale bar = 2 μm.

Originating in the ventricular interior of the brain, nascent CSF is continuously produced by the CPs in the lateral, 3^rd ^and 4^th ^ventricles. Because CNS metabolism is profoundly affected by CSF transport, it is imperative to delineate the physiological and molecular processes in CP that effect CSF secretion. CSF formation occurs in two stages: passive filtration of fluid across choroidal capillary endothelium [25], followed by a regulated active secretion across a single-layered epithelium (Fig. 3). Scores of investigations have characterized solute movement from blood to CSF by diffusion, facilitated transport and active transport. Such studies elucidate the nature and uniqueness of fluid transfer across the BCSFB.

Fluid is generated at a high capacity by the choroid plexus epithelial (CPe) cells [1]. The CPe secretory engine is fueled by a brisk arteriolar input of organic substrates and water to the extensive capillary plexus [1]. A blood flow of about 4 ml/min/g CP is converted to a CSF formation in mammals of approximately 0.4 ml/min/g. The smallest mammalian species in which formation rate has been quantified is the mouse which produces CSF at 3.3 × 10^-4 ^ml/min [26]. If one extrapolates animal data to humans on the basis of CSF produced per volume of brain tissue, human CSF production should be about 0.8 ml/min. Measurements to date, however, show that human CSF production is roughly one-half that rate [3].

CSF formation begins as plasma is filtered across permeable choroidal capillaries [25]. Net filtration is proportional to the hydrostatic pressure gradient between blood and choroid interstitial fluid (ISF). Normally, in accord with Starling's law of filtration, fluid flows freely from plasma into ISF at the basolateral surface of the epithelial cells. Reduced blood flow to the plexus, via rate-limited tissue perfusion, curtails plasma filtration into the interstitium. Consequently in acute hydrocephalus the substantially elevated ventricular CSF pressure, when retrogradely transmitted to choroidal ISF, reduces plasma exudation into ISF. This decreases CSF formation. However with normal choroidal perfusion and CSF hydrodynamics, a continual stream of plasma ultrafiltrate (ions and water) is presented for fluid manufacture to transporters at the basolateral side of the epithelium.

**Figure 4:**
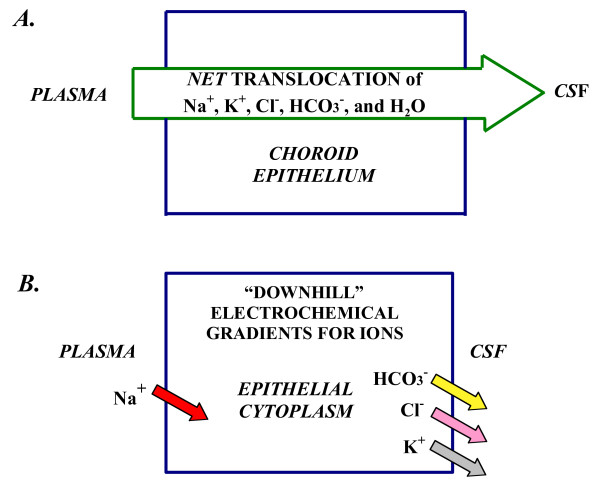
**Mechanisms of CSF formation:** Net ion transport and electrochemical gradients A. CSF secretion by CP is by net transport of Na^+^, K^+^, Cl^-^, HCO_3_^- ^and water, from plasma to ventricles. Reabsorptive ion fluxes CSF to blood occur simultaneously with active secretion but overall, there is net movement of ions and water into ventricles. CSF osmolality resembles plasma. B. Na^+ ^moves down a concentration gradient via secondary active transport (e.g., Na^+^-H^+ ^exchange) in the basolateral membrane (Fig. 5) [38, 43]. K^+^, Cl^- ^and HCO_3_^- ^diffuse down their electrochemical gradients [38] via ion channels in apical membrane [50] (Fig. 5). Arrow for Na^+ ^symbolizes a steep inward concentration and electrochemical gradient [38]. For K^+^, Cl^- ^and HCO_3_^-^, the respective arrows depict outwardly-directed electrochemical gradients promoting ion diffusion via channels into CSF [50]. Choroidal cell concentrations (mM) for Na^+^, K^+^, Cl^- ^and HCO_3_^-^, respectively, are about 48, 145, 65, and 9.5 for rat [38]. The potential difference across the CPe membranes is 45–50 mV, CSF being about 5 mV positive to plasma at pH 7.4.

**Figure 5:**
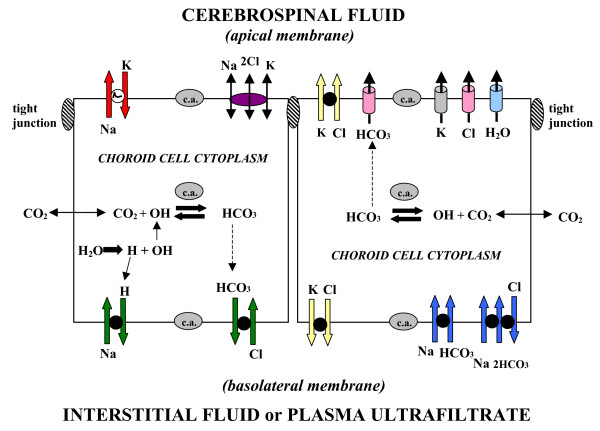
**Ion transporters and channels in mammalian choroidal epithelium.** Typical CPe is schematized to localize transporters and channels that transfer ions and water [290]. CSF secretion results from coordinated transport of ions and water from basolateral membrane to cytoplasm, then sequentially across apical membrane into ventricles. On the plasma-facing membrane is parallel Na^+^-H^+ ^and Cl^-^-HCO_3_^- ^exchange [38] bringing Na^+ ^and Cl^- ^into cells in exchange for H^+ ^and HCO_3_^-^, respectively. Also basolaterally located is Na^+^-HCO_3_^- ^cotransport (NBCn1) [41] and Na-dependent Cl^-^-HCO_3_^- ^exchange [42] that modulate pH and perhaps CSF formation. Apical Na^+ ^pumping [49, 110] maintains a low cell Na^+ ^that sets up a favorable basolateral gradient to drive Na^+ ^uptake. Na^+ ^is extruded into CSF mainly via the Na^+ ^pump [110] and, under some conditions, the Na^+^-K^+^-2Cl^- ^cotransporter [46]. K^+^-Cl^- ^cotransport helps maintain cell volume [50]. Apical channels facilitate K^+^, Cl^- ^and HCO_3_^- ^diffusion into CSF [68]. Aquaporin 1 channels on CSF-facing membrane [77] mediate water flux into ventricles. Polarized distribution of carbonic anhydrase (c.a.) and Na^+^-K^+^-ATPase [51], and aquaporins, enable net ion and water translocation to CSF.

Transchoroidal secretion of water, ions and macromolecules drives the volume transmission of CSF (Fig. 2) down the ventriculo-cisternal axis [27]. Actively formed CSF stems from coordinated secretion of solutes across the thin epithelial interface (Fig. 3) between the inner choroidal plasma and outer ventricular fluid. The CP epithelium is polarized both structurally (Fig. 3) and functionally (Figs. 4 and 5). Pharmacological modeling of CSF dynamics is necessarily built on foundational knowledge of plexus blood flow as well as epithelial metabolism and transport (Fig. 5).

CSF formation involves an intricate array of transporters and channels in CPe. Salient elements of this complex process deserve highlighting. Accordingly, Fig. 4 schematically illustrates how choroid epithelial polarity (sidedness) relates to transcellular movement of solutes across the BCSFB. Although bidirectional transport of ions occurs at both poles of choroid epithelial cells, CSF formation is essentially the net transport of Na^+^, Cl^-^, K^+^, HCO_3_^- ^and water from plasma to CP to CSF (Fig. 4A). Ions and water are taken up by facilitating mechanisms at the basolateral membrane, convected through cytoplasm, and then released or actively secreted into the ventricles on the apical side. Vectorially, favorable electrochemical gradients (energetically downhill) exist for Na^+ ^movement inward at the plasma side and for K^+^, Cl^- ^and HCO_3_^- ^diffusion outward at the CSF side (Fig. 4B). Facilitated water flux via aquaporin (AQP) channels osmotically balances the collective ion transport and furnishes the medium for CSF circulation.

CSF generation is a cardinal feature of brain fluid homeostasis [1]. To finely control the CSF formation rate is a challenge in treating diseases related to brain fluid disorders. Historically, emphasis has been placed on discovering new agents to relieve elevated CSF pressure (CSFP), i.e., intracranial pressure (ICP), in pediatric hydrocephalus patients. Projections point to the additional compelling need for increasing CSF turnover rate in geriatric patients, since many are taking diuretics and digitalis preparations that reduce CSF formation. This implicates a wider spectrum of CSF pharmacology. To individualize therapy for normal-pressure and high-pressure hydrocephalus, there should be a drug repertoire for up- and down-regulating CSF formation rates.

Pharmacological targeting to modify CSF production by the CP will advance as cellular mechanisms are elucidated. To date, acetazolamide and furosemide are among only a few agents that have had clinical usefulness in controlling CSF formation and ICP, but side effects limit their usage [28,29]. Clearly, new agents are needed to restore CSF dynamics altered by disease and injury. Theoretically, nuclear, cytoplasmic and plasma membrane constituents are potential sites for drug actions to accelerate or reduce CSF production. Transcription factors in the nucleus, enzymes in the cytoplasm, and transporters/channels and receptors at the limiting plasma membrane are all potential drug targets. Five approaches to modify CSF formation rate, i.e., cutting-edge as well as established, are recapitulated in Table 2 and elaborated below:

**Table 2:** Strategies for pharmacologically manipulating the rate of CSF formation by the epithelial cells of choroid plexus

- Modulation of transcription factors or nuclear receptors that control expression of enzymes involved in CSF formation
**Molecular targets**: p73, foxJ1, and E2F5 [30, 31]
- Interference with the basolateral and apical membrane-associated ion translocaters (cotransporters, exchangers and pumps)
**Molecular targets**: Na-K-Cl cotransporter [45–48]; Na-H exchanger [36–38]; Cl-HCO_3 _exchanger [40, 55]; Na pump [49, 106, 107, 110] Na-HCO_3 _cotransporter [41]; Na-dependent Cl-HCO_3 _exchanger [42] K and anion channels in apical membrane [68]
- Inhibition of the enzymatic generation of labile ions in cytoplasm and in microdomains of the plasma membrane
**Molecular targets**: carbonic anhydrase isoforms [43, 44, 52, 53, 56–58]
- Regulation of the expression and activity of aquaporin water-conducting channels in the apical (CSF-facing) plasma membrane
**Molecular target**: Aquaporin 1 channel [65–68, 70–78, 292]
- Stimulation of plasma membrane receptors for fluid-regulating neuropeptides
**Molecular targets**: V1 receptor [83, 84, 90, 207, 210, 217]; NPR receptors [85, 97, 101, 103]; AT1 receptor [217]

### 2.1 Transcription factors

Traditionally CSF formation has been experimentally altered by interfering with membrane ion transporters or by reducing delivery of substrate. Nevertheless, metabolically upstream molecular manipulations for modulating CSF secretion deserve consideration. A novel approach is to target transcription factors as putative regulators of cellular enzymes in CSF formation. Mice lacking the transcription factors p73, foxJ1 and E2F5 develop non-obstructive hydrocephalus [30,31], possibly from CSF over secretion. Epithelial ultrastructure analysis and electron translucence in micrographs are consistent with accelerated fluid formation by CP [32]. It is intriguing that E2F5 in CPe is down-regulated as the cells mature (i.e. as they convert from pseudostratified to single cuboidal epithelium) in their ability to produce CSF [30]. Such ontogenetic findings on transcription factors in mice and human CP [30,31] prompt a new tack to augment CSF formation by manipulating E2F5 expression.

Releasing the natural inhibitory brake on the manufacture of CSF would be a unique way to increase fluid transfer across CP. There is an experimental precedent for this theoretical approach. Resection of the sympathetic nerve from the superior cervical ganglion attenuates the inhibitory autonomic tone on CP. This increases CSF production [32]. Enhancing CSF formation and the associated sink action [33] on brain interstitial solutes may find application to aging, normal pressure hydrocephalus (NPH), and Alzheimer's disease (AD) patients who retain extracellular metabolites from sluggish fluid turnover [19].

### 2.2 Ion transporters

Most attempts to regulate CSF formation rate have been directed at altering ion movement via transporters in the basolateral (plasma-facing) and apical (CSF-facing) membranes of the CPe [1]. Because the CP is a kidney-type organ [34], with epithelial mechanisms [35] similar to those in the renal tubule that transfer a large volume of ions and water, many diuretic agents have been tested for ability to inhibit CSF formation [1,27]. Gaining insight on the many ion transporters and channels in CPe is key to attaining fine control of CSF production, upward as well as downward regulation.

At the basolateral membrane, the uptake of Na^+ ^and Cl^- ^is the primary step in CSF formation (Fig. 5). Inward transport of fixed ions, Na^+ ^and Cl^-^, is facilitated by carriers functionally dependent upon basolateral transmembrane gradients for Na^+^, Cl^-^, H^+ ^and HCO_3_^-^. The basolateral Na^+^-H^+ ^exchanger (NHE1) transfers Na into the choroid cell. This process is inhibited by the diuretic agent amiloride [36-38], which decreases CSF formation [36,38]. This action is analogous to the polyuria caused by inhibiting the proximal and distal tubule Na^+^-H^+ ^exchanger (NHE3) that normally supports substantial fluid transfer in kidney [39]. The Cl^-^-HCO_3_^- ^exchanger translocates interstitial Cl^- ^into the cytoplasm. Moreover the basolateral Na^+^-dependent Cl^-^-HCO_3_^- ^exchanger and the Na^+^-HCO_3_^- ^cotransporter in CPe [40-42] may also be potential targets for controlling fluid movement. Na^+ ^coupling to HCO_3_^- ^transport is likely integral to nascent CSF generation [43,44]. Systemic metabolic alkalosis, in comparison to metabolic acidosis, promotes Na^+ ^influx into CPe and subsequently into ventricular CSF [38]. Therefore basolateral HCO_3_^- ^transport [40,42] by one or more mechanisms (Fig. 5) might be manipulated to modify HCO_3_^- ^flux or alter choroid cell pH to modulate fluid formation.

At the apical membrane Na^+^-K^+^-ATPase, the Na pump, is a workhorse that actively secretes Na into the ventricles (Fig. 5) thereby driving CSF formation. The Na pump critically keeps cell Na low [38] to set up a driving force (favorable gradient) for basolateral inward Na movement via the Na-cotransporters. Therefore, pharmacological disruption of the apical Na pump, by distorting transmembrane ion gradients, secondarily disables transport activity at the opposite basolateral face. The apically located Na^+^-K^+^-2Cl^- ^cotransporter (NKCC1) is significant in regard to bidirectional transport capability. The ability of the cotransporter to transfer ions in both the secretory and reabsorptive modes [45] reflects a versatile transporter for regulating cell ion homeostasis and CSF dynamics. Interference with NKCC1 activity by bumetanide [46] or furosemide reduces CSF formation rate [47]. In both congenital [48] and aging [18] disorders, altered expression of NKCC1 in CPe is associated with diminished CSF formation rate. Drugs used in the aging population, e.g., the loop-diuretic furosemide and the cardiac glycosides (that inhibit the choroidal Na^+ ^pump [49]), reduce CSF formation rate and may therefore exacerbate the deleterious effects of advanced age on the brain. Research is needed to address how apical membrane transporters, as well as ion channels for K^+^, Cl^- ^and HCO_3_^- ^[50], can be manipulated to increase or decrease CSF formation rate, depending upon specific clinical needs.

### 2.3 Enzymes that modulate transport

In experimental settings, Na^+^-K^+^-ATPase and carbonic anhydrase (c.a.) are the two enzymes in the CPe that have been the most extensively analyzed in inhibitory studies of CSF formation. Na^+^-K^+^-ATPase, or the Na^+ ^pump, is atypically (amongst epithelia) located on the luminal or CSF face of the CPe. Therefore ouabain inhibits the Na^+ ^pump (which is integral to CSF formation) when placed intraventricularly, but not intravenously. In addition to interfering with CSF production, the Na^+^-K^+^-ATPase inhibition also alters CPe volume, potential difference and ion homeostasis [51]; therefore, ouabain and other cardiac glycosides have limited value in regulating human CSF formation. On the other hand, the inhibition of c.a. by acetazolamide is not as harsh on CPe function, and so can be used therapeutically to reduce CSF generation. Because a variety of c.a. isoforms is expressed in CP, there are potentially multiple targets at the blood-CSF interface for regulating fluid dynamics.

Ubiquitous enzymes in the c.a. group integrate cellular acid-base phenomena with CSF formation [52]. One of the earliest, but still clinically useful, means for decreasing CSF formation is to reduce the intracellular generation of labile ions (e.g., protons and bicarbonate) that feed fluid transporters at the plasma- and CSF-facing poles of the CPe. Cytosolic c.a. II catalyzes cellular hydration of CO_2 _to yield H^+ ^and HCO_3_^-^. Acetazolamide, a sulfonamide drug, slows down this reaction [44]. Consequently this elevates choroidal cell pH, thus reducing H^+ ^availability to basolateral Na^+^-H^+ ^exchange [43,53,54]. Membrane-bound isoforms of c.a. (e.g., c.a. VIII) are likely proximate to basolateral HCO_3_^- ^transporters (e.g., anion exchanger 2 or AE2) to which they supply HCO_3_^- ^for exchange with interstitial Cl^- ^[55]. It is worthwhile seeking non-sulfonamide agents to selectively inhibit specific c.a. isoforms, II, VIII and XII [56-58]. Identifying a drug for a specific line of attack [59] may help to reduce systemic side effects that attend chronic acetazolamide administration.

Disruption of cellular acid-base balance by diminishing c.a. activity with acetazolamide provides deductive insights on the function of transporters and aquaporins. CPe pH_i _increases by 0.3 – 0.4 pH units after acute exposure (1 hr) to acetazolamide [43,53]. Chronic administration of acetazolamide markedly alters CP function and structure [60], including the apical microvilli which contain the AQP1 channels. In the kidney, and in the testis (another barrier tissue), acetazolamide down-regulates AQP1 expression [61-64], possibly by way of pH_i _change. With regard to fluid transfer in the kidney, the crucial importance of Na^+^-H^+ ^antiport exchange (via NHE3) and pH_i _stability for maintaining intact AQP2 and NKCC2 systems is revealed in NHE3-knockout analyses [39]. In the physiologically analogous CPe, the coordination of multiple transporter systems (NHE1 and NKCC1) with AQP1 channel function evidently allows CPe ion and volume homeostasis to occur simultaneously with CSF secretion [38].

### 2.4 Aquaporins or water channels

Yet another potential avenue for regulating CSF formation exploits the expression plasticity of aquaporins (AQP) in CP [65,66]. Aquaporin channels, which facilitate water diffusion across the blood-CSF interface [67-69], appear in the developing CPe [70,71] and are retained throughout life. AQP1, initially designated as CHIP28 [72], is heavily expressed at the ventricular-facing membrane of CPe [73,74] but not in blood-brain barrier (BBB) endothelium. Thus by regulatory phosphorylation or ubiquitination of AQP1 [75], it may be possible to selectively modulate water movement from blood into the cerebral ventricles [76]. In AQP-1 null mice, there is a substantial reduction in CSF forming-capacity of the CP; and consequently, an associated lowering of the CSFP [77,78]. Such knock-out studies imply that the number of AQP1 channels in CPe apical membrane importantly determines CSF manufacturing ability.

Of significance to CSF dynamics, nature performs an experiment in which there is AQP1 deficiency in late life [57]. Thus, aged (20-mo) Sprague-Dawley rats have substantially less AQP1 expression in CPe than do young adult counterparts [57]. Accordingly, senescent rats are less able to form CSF [79]. Human CPe expresses AQP1 [80]. This raises the possibility of therapeutically upregulating or restoring AQP1 expression in aged humans when the CSF turnover rate is compromised by AD and NPH [3,19]. Conversely in congenital hydrocephalus it may be feasible to down-regulate AQP1 expression or channel activity in CPe, thereby lowering CSFP by suppressing CSF formation rate [77,78]. Atrial natriuretic peptide (ANP), a CSF-modulator, has been implicated as a regulator of AQP1 channel function [69].

### 2.5 Receptors for neuropeptides

Neuropeptides in CSF stimulate peptidergic receptors in CP to alter metabolism of 2nd messengers and the associated ion transport coupled to fluid formation [81]. Choroidal arginine vasopressin (AVP) is functionally interactive with various fluid-regulating peptides (Table 3). AVP release from CP, for autocrine inhibitory regulation, is promoted by angiotensin (Ang II) and basic fibroblast growth factor (FGF2). Upon stimulation, choroidal receptors for ANP and AVP both induce dark epithelial cells [82,83]*in vitro *as well as *in vivo*. Dark CPe cells have a neuroendocrine role to down -regulate CSF production. Stimulation of V1 vasopressinergic receptors induces dark cells [83], reduces Cl^- ^efflux from CPe [83], and decreases CSF formation [84]. Moreover, ANP transcripts for three natriuretic receptors [85] that generate cyclic guanylyl monophosphate (cGMP) [86] are expressed in both intact and cultured CP. In some physiological settings ANP curtails CSF formation by CP [87]. This inhibition may be mediated by CPe dark cells [82] that are more frequent in hydrocephalus.

**Table 3:** Neuropeptides/receptors that regulate CSF formation

	ANP	AVP	ANG II	FGF2
Effect on CSF formation after i.c.v. administration of neuropeptide [84, 87, 178, 217]	↓	↓	↓	↓
Peptidergic effect on choroidal blood flow [84, 139]	↑	↓	↓	N/A*
Inducer of neuro-endocrine-like dark epithelial cells in choroid plexus? [82, 83, 178]	Yes	Yes	N/A	Yes
Choroid epithelial receptors for peptides [83, 85, 103, 211, 217]	NPR-A	V1	AT1	FGFR2
Concentration of peptide in CSF in hydrocephalus or increased ICP [98–100, 104]	↑	↑	N/A	N/A

* N/A: Data are not available.

Also supporting this fluid homeostasis model are the observations of substantial plasticity of choroidal receptors for water-regulating neuropeptides when CSF dynamics are imbalanced. There is up-regulated or down-regulated density of CP receptors for ANP in hydrocephalus [88], depending upon the pathophysiology (kaolin-induced as well as congenital). CSF neuroendocrine regulation is also reflected by changes in ANP receptor density secondary to altered CSF hydrodynamics or fluid shifts in space flight [89]. Moreover there are more choroidal binding sites for AVP in AD [90], a disorder that can present with ventriculomegaly [3] and increased CSF pressure [91]. Peptide analogs of ANP and AVP [17] may prove beneficial for therapeutically modulating CSF parameters through action at CP neuropeptide receptors.

## 3 CSF pressure

CSFP or ICP rises rapidly when there is a significant imbalance between CSF formation and drainage. Augmented ICP reduces cerebral blood flow (CBF) and oxygenation and, at the molecular level, may alter protein expression in neurons and glia. Therefore, because of potentially devastating effects of elevated ICP on brain functions, it is clinically important to assess factors that regulate CSF pressure and devise more effective ways to monitor and manage distorted ICP.

### 3.1 Servomechanism regulatory hypothesis

ICP is a complex function of hydrodynamic and hemodynamic parameters. In combination they produce, in adult humans, a CSFP of 100–200 mm H_2_O measured in the lateral recumbent position by lumbar puncture. This pressure is directly proportional to the CSF production rate and outflow resistance, which typically is 70 mm H_2_O/ml/min and greater in the 8^th ^decade than in young adults [92].

On the hydrodynamics side, feedback down regulation of CSF formation can lower CSFP. Homeostatic adjustments to decrease fluid output by CP are critical because sustained CSFP elevation compromises the vascular supply to the CNS [93]. Accordingly, elucidation of neuroendocrine fluid regulation is essential for developing agents to manage CSFP. A key observation for constructing a CSFP regulatory model is that the CSF concentration of most neuropeptides, including ANP, depends upon a central (CNS) source rather than a peripheral (plasma) source [94]. Moreover, intraventricularly-administered ANP reduces CSFP elevated by ischemia [95] or congenital hydrocephalus [96]. Such findings prompt the pursuit of various elements comprising a central homeostatic system to regulate CSFP.

Growing evidence supports a ventricular servomechanism to stabilize CSFP. One hypothesis posits pressure-sensitive regulatory elements in the CSF-periventricular nexus that respond by synthesizing/releasing peptides that target the CP [97]. A prime neuropeptide candidate for acutely regulating CSFP is ANP. Collective experimental and clinical evidence demonstrates that: i) the CSF titer of ANP increases proportionally to CSFP elevation [98], ii) the CSF level of ANP is elevated in high-pressure hydrocephalus [99,100], iii) the ANP receptors in CP undergo a Vmax adjustment to compensate for the CSFP increase in hydrocephalus [88,101], and iv) the CPe is bioreactive to ANP [82] and modulates CSF formation [102] by a cGMP-dependent mechanism [86,103]. The putative regulation of CP-CSF by ANP and AVP occurs in acute high-pressure hydrocephalus but evidently not in chronic NPH [104]. Interestingly the CP of immature rats (with a relatively low CSFP and incompletely-developed CSF secretion [33]) is less sensitive than the adult epithelium to modulation by ANP and AVP [83]. This is probably due to infant vs. adult differences in neuropeptide receptor density and coupling to CPe metabolism.

### 3.2 Ontogeny of CSF pressure generation

Increasing CSFP during development in rodents is temporally associated with the early phases of fluid formation by CP. CSF pressure in pentobarbitone-anesthetized exteriorized fetal rats (18–21 days p.c.) is about 20 mm H_2_O, rising to 34 mm H_2_0 at 10 days postnatal and later [105]. The 1 – 3-week postnatal interval in the rat is a watershed period in the maturation of CP-CSF secretion [33,106]. Thus the increasing vascularity and arterial perfusion of the plexus in early postnatal life [107-109] foster the progressively increasing enzymatic and transport capabilities of the CPe [110,111]. Enhanced blood flow and metabolic rate combine to accelerate Na^+ ^and Cl^- ^transport into ventricles [112]. Such incremental fluid production by CP [113] elevates CSFP to a level found in adults [105]. Arterial pulsations in CP along with increasing fluid turnover set up a pressure head for moving CSF down the neuraxis [114] and developmentally opening up subarachnoid sites for reabsorption. This increased hemodynamic-hydrodynamic generation of CSF secretion and pressure pulse fosters a spurt in brain growth [106-113]. Maintaining appropriate CSFP in the developing CNS critically depends upon aqueductal patency and smooth CSF flow. Just as intraocular pressure importantly molds the shape of the developing eye, the typically-moderate rise in CSFP in perinatal stages of life [105] contributes to morphing the expanding CNS.

### 3.3 Congenital hydrocephalus and periventricular regions

Untoward effects on brain development, however, result from inordinately elevated CSFP secondary to blockage of the Sylvian aqueduct. Augmented CSFP has been observed in aqueduct-impaired hydrocephalic (H-Tx) rats, increasing 2-fold between 10 and 21 days after birth [115]. In other H-Tx studies, CSFP increased from about 20 mm H_2_0 at 10 days after birth to as high as 90 mm H_2_0 by 3 weeks [116,117]. Elevated pressure reflects blocked flow out of the lateral ventricle. The consequent CSF stasis in hydrocephalus interferes with cerebral and ventricular system development [118,119].

Transgenic mice lacking ependymal-specific expression of hydin [120], which facilitates ciliary functions, have gross distortions in the anatomical relationship between the ventricles and adjacent brain. Certain hydrocephalic malformations stem from metabolic, transport and ciliary deficiencies in the developing CP [121]. Systematic investigations should explore how faulty cilia development in the choroido-ependymal lining affects CSF formation, composition, volume and ultimately the CSFP.

CSFP impacts periventricular protein expression and secretion into the ventricles. An optimally-growing CNS depends on delicately-balanced biophysical and biochemical phenomena in CSF [6]. In congenital hydrocephalus, however, there is not only increased pressure but also altered CSF composition [118,119,121]. Nerve growth factor concentration is elevated in CSF of children with congenital hydrocephalus [122], but cytokine levels are evidently stable (implying intact BBB function) [123]. Whereas some alterations in CSF constituents are due to interrupted CSF flow [119,121], other changes in composition are related to byproducts of pressure-induced oxidative damage to cells near the ventricles [124]. Other modifications of CSF chemistry in hydrocephalus, possibly homeostatic, may be due to pressure-induced up-regulation of proteins secreted by CPe, ependyma and periventricular tissue. One example is choroidal growth factor synthesis in response to augmented CSFP that follows ischemia [7] or trauma. CPe and ependyma are a plentiful source of growth factors [125,126] to repair tissue injured by elevated CSFP and its secondary effects [8].

The interaction between the pressures of the CSF and vascular systems, especially in the onset of hydrocephalus, awaits systematic investigation. Fascinatingly, the elevated arterial pressure in spontaneously hypertensive rats is linked to ventriculomegaly via effects on secretion by the subcommissural organ (SCO) in the aqueductal wall [127]. The SCO and its associated Reissner's fiber (RF) secrete into CSF glycoproteins essential for establishing normal CSF flow down the neuraxis for distal reabsorption. Therefore, a disrupted SCO-RF complex results in less efficient CSF flow and consequent hydrocephalus [128,129]. Compromised function of the SCO [130] as well as CP [121] leads to ventriculomegaly and imbalanced CSF dynamics. These observations point to the significance of the circumventricular organs in secreting factors to optimize developing CSF flow, volume and pressure.

### 3.4 Brain response to elevated CSF pressure

In addition to the pressure effects on the periventricular zone, there are several ways that CSFP impacts the brain globally. Responses to elevated CSFP can be broadly categorized by: i) how neural structure and metabolism are affected by modified CSF dynamics; and ii) how homeostatic transport interfaces that protect the parenchyma respond to neuropeptide up-regulation in CSF hypertension. For the first consideration, there are marked oxidative changes [131-133] in hydrocephalus that are reflected in the way that injured neurons metabolize neurotransmitters [134] and myelin [135]. Although beyond the scope of this review, the topic of deleterious anaerobic neuronal metabolism in hydrocephalus has been treated previously [136-138]. Concerning the second consideration, i.e., homeostatic mechanisms, the regulatory increases in ANP and AVP concentrations in CSF from elevated CSFP [98-100,104] evidently foster vascular perfusion of several regions. Even though up-regulated ANP may decrease CSF formation in ischemia and hydrocephalus, it does support CPe viability by increasing choroidal blood flow [139]. CSF-administered ANP and AVP dilate cerebral arteries [140,141]. ANP- and AVP-dilating effects on large pial arteries thereby counter cerebral vasospasm induced by endothelin following subarachnoid hemorrhage [142]. Such perfusion-supporting actions by centrally up-regulated ANP curtail edema [143] by stabilizing the CP and cerebral vasculature threatened by ischemia and augmented CSFP.

**Figure 6:**
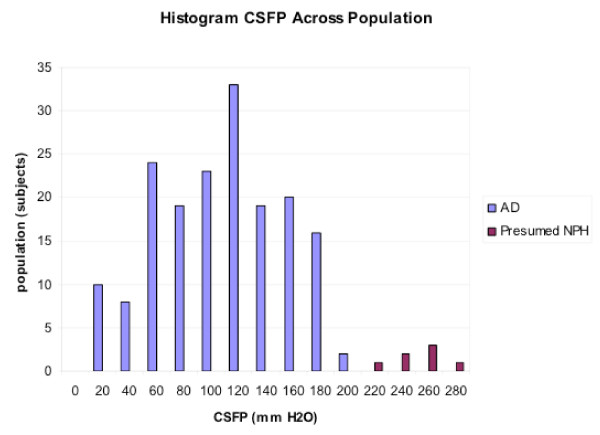
**Frequency analysis of CSFP in patients with AD vs. AD-NPH syndrome.** AD subjects by NINDS-ADRDA (Alzheimer's Disease and Related Disorders Association) criteria (n = 222) were initially screened to exclude NPH. 181 of these 222 patients had CSF pressure measurements (supine position). Seven subjects (4%) had a CSFP 220 mmH_2_O or greater, i.e., higher than the mean CSFP of 103 mmH_2_O for the AD-only group. The 7 subjects with elevated pressure had NPH as well as AD, i.e., the NPH-AD syndrome. The larger CSFP peak corresponds to the AD-only group; the smaller peak (higher pressures) pertains to AD-NPH hybrids. Reproduced from Silverberg *et al *[91].

In chronic hydrocephalus, even moderate increments in CSFP can compromise CBF and metabolism. Intermittent changes in CSFP need a thorough analysis, particularly in regard to effects on regional hydrodynamics and hemodynamics. Clearly, prospective as well as retrospective studies [91] on CSFP are essential to advance treatment strategies for CSF diseases (Fig. 6).

### 3.5 Advances in measuring CSF waveforms

CSFP wave analysis provides substantial insight on the state of CSF dynamics and is more reliable than assessments of cerebral perfusion pressure and mean ICP, especially when there are differences in baseline pressure [144]. CSF has a characteristic pressure pulse that varies in health and disease. Cardiovascular parameters markedly affect CSFP waves by contributing systolic and diastolic components of pulse amplitude and latency [145-148]. Arterial pressure pulsations are transmitted across CPs to ventricular fluid [114] and generate CSF pressure waves. It would be clinically useful in hydrocephalus [149] to characterize these pressure waves in the ventriculo-subarachnoid system.

How is ICP recorded, and does it matter where sensors are placed, i.e., in the CSF, brain parenchyma or epidural space? Continuous ICP signals have been recorded with sensors such as the Camino OLM ICP and Codman ICP MicroSensor [144]. A study of brain parenchyma vs. epidural space monitoring revealed marked differences in recorded mean pressure, but not in the parameters of mean ICP wave amplitude and mean ICP wave latency [150]. Thus, epidural ICP recording (to estimate parenchymal pressure) is feasible for determining parameters such as single wave pulse pressure amplitude (dP) and single wave latency (dT, or rise time). Moreover, monitoring ICP in the cerebral ventricles, as waveform parameters, gives comparable results to pressure measurements done with the sensor in brain parenchyma [145]. Evidence is lacking for an appreciable pressure gradient between the parenchyma and ventricular CSF.

**Figure 7:**
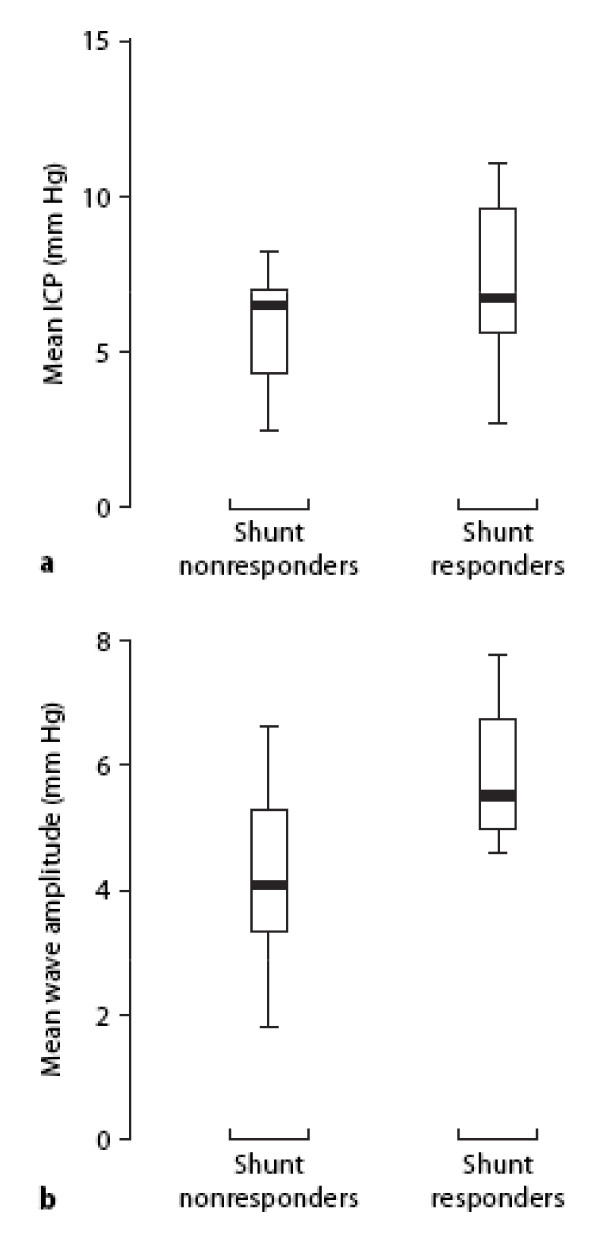
**Augmented intracranial pressure wave amplitude in shunt responders:** The difference between shunt non-responders and shunt responders (i.e., improved CSF dynamics and brain function) 6–9 months after surgery with regard to preoperative mean ICP (*P *= 0.19) and mean ICP wave amplitude (*P *= 0.002; based on 1-way ANOVA of 6-sec time window data). Mean wave amplitude was 5 mm Hg in positive responders. Reproduced with permission from P. Eide [157].

Pulsatile components of CSFP will likely be more helpful than mean CSFP in evaluating intracranial compliance (Fig. 7). Eide introduced a method for continually recording ICP signals at 100 Hz, converting to digital data, and then processing in 6-s time windows [151]. An algorithm filters noise prior to accepting cardiac beat-induced single ICP waves for analysis of amplitude and latency. Wave amplitude analysis is also promising in pediatric hydrocephalus [152] because it is a better predictor of intracranial compliance than is mean pressure. Children with intracranial hypertension from hydrocephalus or craniosynostosis had a more successful outcome after surgery if the mean CSF wave amplitude pre-surgery was > 5 mm Hg [152]. To assure quality control of the waveforms processed, an algorithmic analysis screens out infarct-induced waves to improve diagnostic information [153].

ICP/CSF waveform analyses have predictive potential for outcome in adult patients with chronic hydrocephalus. Although patients with idiopathic NPH (iNPH) may have normal mean ICP, it is possible that they have altered single ICP waves. Higher values of mean ICP wave amplitudes were found in iNPH patients who eventually improved after shunting [154]. In one cohort, the mean CSFP wave amplitude was a better predictor than resistance to CSF outflow (Ro), in regard to positive clinical change a year after shunting [155]. Up to 60% of iNPH patients have augmented mean CSF wave amplitudes, and 90% of these individuals respond favorably to shunting [156]. Post-shunting cognitive outcomes [157] were better in iNPH patients with relatively high pre-operative mean ICP wave amplitudes (Fig. 7).

The so-called B waves in CSF, especially in NPH patients, can predict post-surgical outcome in adult chronic hydrocephalus. Slow B waves, however, only weakly correlated with improvement after shunt surgery [158]. An alternative to B wave examination has been proposed. Accordingly, the relative pulse pressure coefficient (RPPC) combines the effects of pulsatory volume changes and elastance on CSFP [159]. RPPC reflects reallocation/storage rather than reabsorption of CSF because it relates pulse amplitude to mean CSFP.

A worthy clinical goal is to ascertain absolute CSFP by methodology minimally disruptive to CSF or brain. CSF is contiguous with fluids in the ear, nose and eyes. The orbit vasculature, for example, is exposed to ambient CSFP. This should enable reliable CSFP determination from transocular sonographic and dynamometric data. An empirical index based on ocular arterial (blood flow) and venous (outflow pressure) measurements highly correlates (r = 0.95) with absolute CSFP [160]. Therefore this promising indirect ocular approach deserves refinement.

Technical advances are also being made with directly measured CSFP. A novel technique minimizing CNS invasion utilizes a hollow mandrin. This ventricular probe allows precise cannulation of CSF and determination of opening CSFP without fluid loss along the penetrating catheter [161]. This refined mandrin approach consistently accesses the ventricles with a single puncture compared with occasional multiple penetrations in previous cannulation procedures to locate the CSF [161]. Overall, the aim of CSFP assessments is reliability by minimally perturbing the CSF-brain.

To expedite CSF and hydrocephalus modeling, attention is being devoted to delineating CSFP signals. Highly precise signals for hemodynamic and hydrodynamic parameters facilitate CSFP analyses. Continuous signal inputs to multiple sensors (blood pressure and CSFP during infusions) are integrated by software [162] to evaluate fluid dynamics and predict steady-state responses to infusion tests. Computerized rheoencephalography non-invasively assesses NPH by analyzing changes in cerebral electrical impedance related to pulsatile blood flow [163]. Arterial compliance determined by rheoencephalography is evaluated from the regression of arterial pulse amplitude (aAMP) on mean CSFP. Patients with lower arterial compliance have higher slope values for aAMP vs. CSFP. Increasingly sophisticated analyses of CSFP against arterial pressure will improve predictive shunting. Models of fluid-structure interactions to predict pressure and flow within the ventricles [164] and transmittal of pressure into brain parenchyma [165,166] are advancing the theory of pulsatile fluid dynamics.

## 4 CSF flow

CSF flow pathways need elucidation for both healthy and diseased brain. Choroidally secreted CSF flows down the neuraxis to the 4^th ^ventricle and then out through hindbrain foramina into the cisterna magna and the subarachnoid space in basal regions. In addition to this classically-described pathway, new evidence indicates that ventricular CSF also flows by another route to the basal and midbrain cisterns, i.e., into subarachnoid extensions [167] of the velum interpositum (from dorsal 3^rd ^ventricle) and superior medullary velum (rostral 4^th ^ventricle). Additional evaluation is needed for the limited CSF flow from cisterna magna to cortical subarachnoid regions and arachnoid villi [168], and for the possible shunting of CSF bulk flow between the ventricles and injured/edematous brain parenchyma [169]. Insight gained on CSF distribution routes will benefit pharmaco-therapeutic regimens.

### 4.1 CSF flow and brain metabolism

There is increasing appreciation that reduced CSF flow adversely affects brain metabolism and fluid balance [3,18,19,118,119]. Historically in hydrocephalus investigations, CSF pressure and volume [170-173], not flow and composition, have been emphasized in animal models and shunt design. Consequently, considerable knowledge has accrued on pressure-volume relationships in the ventriculo-subarachnoid system. Evidence continues to accumulate, however, for the importance of CSF flow on cerebral metabolism. Because the CP-CSF supplies micronutrients and peptides to neuronal networks, and removes many catabolites, impeded CSF flow disturbs metabolism in early life [119] as well as in late stages [174].

### 4.2 Flow effects on fetal germinal matrix

Interference with CSF flow through the ventricles and aqueduct in fetal life profoundly retards brain development [118,119]. This is due partly to compromised transport of growth factors from secretory sites such as CP to subventricular germinative zones where stem cells become neurons. In the H-Tx hydrocephalic rat there is a congenital defect leading to tissue dysgenesis in the aqueductal region [175,176]. The H-Tx model [117-119] displays prenatal ventriculomegaly and distortions in CSF growth factor delivery via volume transmission even before intraventricular pressure rises. Prenatally-altered CSF flow thus causes maldevelopment in H-Tx due to faulty differentiation of neural precursor cells [118]. Because CP function is fundamental for CNS development [2], it is pertinent to assess how perturbed CSF flow and composition harm the growing brain. Non-invasive quantification of aqueductal CSF flow [177] during perinatal life would enhance insights into pediatric hydrocephalus.

### 4.3 Decreasing CSF flow in aging CNS

CSF flow disruption in adult chronic hydrocephalus also devastates cerebral functions. Throughout aging, the ability of CP epithelium to manufacture CSF undergoes continual decline [2,3,57,79]. As CSF formation rate dwindles by 50% or more in senescence and disease [3,19], the sink action [33] of slower-flowing CSF is attenuated [9]. The concentration of potentially-toxic peptides and organic metabolites in CSF [174] and brain consequently builds up due to sluggish flow. Less favorable concentration gradients for catabolites diffusing from ISF to ventricular CSF, results in reduced clearance of harmful substances from brain. Pharmacological studies in old animals treated with flow inhibitors, e.g., acetazolamide [36,53], FGF2 [178,179], and kaolin [180] are needed to analyze the time course of elevated CSF protein concentration in acute vs. chronic states of curtailed flow.

### 4.4 Refinement of non-invasive flow measurements

Due to previous technological limitations, there has been a paucity of investigations to assess CSF flow in animal models. However several non-invasive studies involving magnetic resonance imaging (MRI) to quantify CSF flow have been carried out in humans. One of the earliest such analyses by Ridgway et al. [181] demonstrated that CSF flow is pulsatile, i.e., moving to and fro, alternately in cephalic and caudal directions. Thus, they used the MRI pulse sequence (gated from the R-wave of the electrocardiogram) to calculate CSF flow velocities (e.g., 2.9 cm/sec) and corresponding flow rates (e.g., 0.4–0.6 ml/min). Similarly, dynamic MRI of CSF flow, using a multi-slice spin echo approach, was used to compare aqueductal CSF flow rates before and after ventriculostomy to repair non-communicating hydrocephalus [182]. MR phase imaging has also been used to demonstrate a circadian variation in human CSF flow, the latter being twice as great at night as in daytime [183].

Recent advances have been made in the technology and algorithms for quantifying CSF flow non-invasively. Using phase-contrast MRI to calculate CSF and CBF curves over the cardiac cycle, Stoquart-Elsankari *et al*. [184] extracted data for several CSF parameters: mean and peak flows, latencies and stroke volume. They found that CSF stroke volume was reduced in the elderly, both at the aqueductal and cervical levels. Another new approach is the phase contrast balanced steady-state free precession (PC-bSSFP). It is advantageous over the gradient echo in that signal-to-noise ratio is improved and non-laminar CSF flows can be more efficiently analyzed [185]. PC-bSSFP has been more suitable than the gradient echo approach for quantifying turbulent CSF flow in the prepontine cistern and cervical subarachnoid space [184,185]. Application of refined imaging procedures to small animal models will be helpful in modeling flow pulsatility and the intra-cardiac cycle displacements of CSF volume.

## 5 CSF volume

Maintaining normal CSF volume is indispensable to brain health. CSF volume ranges from the greatly reduced spaces in slit ventricle syndrome to the ventriculomegaly of severe hydrocephalus. The dynamics of ventricular contraction and expansion are poorly understood but likely involve the interplay of three main factors: changes in brain tissue properties (e.g., compliance), CSF dynamics and vascular parameters. CSF volume can be significantly altered iatrogenically by suboptimal rate of CSF flow through shunts. Disorders that rupture the BBB and BCSFB also modify ventricular volume. Understanding CSF volume is important because of the impact on neuronal function exerted by changes in cerebral metabolism and blood flow. Therefore, information is needed on the rate and extent of regional CSF volume adjustments.

Maintaining the appropriate size of the ventriculo-cisternal-subarachnoid compartments is biophysically and biochemically complex. CSF volume in adult mammals ranges from about 0.04 ml in mice [77] to approximately 150–160 ml in humans [3]. Over an organism's lifespan, the CSF volume is variably set to a higher or lower degree in response to stressors. Hypoperfusion of the plexuses [186-188] interrupts the O_2 _supply and severely compromises the BCSFB and ventricular integrity. Hyperthermia also devastates the CP-ventricle nexus. By way of osmotic and autonomic disruptions between the CP and CSF, hyperthermia causes an edematous expansion of the CNS parenchyma [189]. On the other hand, ventricular volume can contract as occurs in slit-ventricle syndrome [190]. Transgenic, hydrocephalic and hypertensive models inform on ontogenetic, physical and physiologic factors that alter ventricular volume. The discussion below treats the overlapping actions of vascular, hydrodynamic and neuroendocrine phenomena in regulating ventricle size.

### 5.1 Hemodynamic factors

CSF volume importantly adjusts to physiological variations in cerebral blood volume (CBV). Thus an increased amount of blood within the cerebrovascular system leads to a displacement, and to a reduced volume, of CSF. Conversely, a decrease in CBV can result in an augmented volume of CSF. This more-or-less reciprocal relationship between CBV and CSF volume reflects the fact that, according to the Munro-Kelly doctrine [191], the combined volume of blood, CSF and brain remains relatively constant in the intracranial space bounded by the rigid skull. Consequently, in order to regulate pressure within the CNS, the CSF is a useful physical buffer that helps to accommodate changes in brain blood supply.

Physical aspects of choroidal hemodynamics also significantly affect CSF volume. There is a brisk, pulsating blood flow of 3–4 ml/g/min to the plexus [108,109]. This strong arterial pulse is transmitted across the choroidal epithelial membranes to CSF. Therefore nearly all CSF motion is pulsatile. It has long been known that CSF dynamics are markedly impacted by vascular pressure waves in CP [114]. Hyperdynamic upstream CP pulsations dilate the cerebral ventricles when there is increased impedance to the flow of CSF pulsations in the SAS [192]. As downstream CSF impedance rises, there is redistribution of CSF pulses from the subarachnoid compartment back to the ventricles and the capillary-venous circulation. Egnor and colleagues propose that communicating hydrocephalus with ventriculomegaly results from pulse redistribution within the cranial cavity [192].

### 5.2 Hydrodynamic factors

Hypo-secretion of CSF, presumably occurring consequent to disrupted bicarbonate transport in CP of SLC4 knockout mice, drastically reduces ventricular volume [193]. On the other hand, hypersecretion of CSF by an actively metabolizing CP papilloma [194], predisposes to ventricular expansion. Thus, altered transport and permeability at the choroidal blood-CSF interface significantly bears on the ventricular volume.

The spontaneously hypertensive rat model (SHR) also reveals the significance of BCSFB transport and fluid formation in regulating fluid volume within the CNS interior. SHR displays a ventriculomegaly [127,195] that likely results from markedly transformed CP functions. Ions, water and non-electrolytes more readily penetrate the blood-CSF barrier of SHR compared with non-hypertensive Wistar-Kyoto (WKY) controls [196]. The CPe of SHR rats consistently has a greater number of mitochondria, increased Golgi processing and enhanced transport activity [197]. The higher permeability of the blood-CSF interface in SHR allows greater penetration of organic solutes such as sucrose from blood to CSF [196]. Increased activity of the Na^+^-K^+^-2Cl^- ^cotransporter [198] which promotes fluid transfer in CP [1], also occurs in SHR. Greater permeability and secretion at the CP in SHR would promote faster transfer of fluid into the ventricles. This is consistent with the ventricular expansion in SHR [127,195]. According to the Egnor model [192], the larger pulse amplitude of the transmitted arterial pulse in the SHR would also be a factor causing ventriculomegaly.

Other injury models of the blood-CSF barrier shed light on dysregulated ventricular volume. Acute arterial hypertension in adult rats transiently opens the BCSFB allowing plasma proteins to leak into the ventricles [199]. Pathological disturbances that enhance CP permeability lead to macromolecule accumulation in the normally low-protein CSF. This increases the osmotic pressure of CSF and draws water into the ventricles. Transient forebrain ischemia also disrupts choroidal ultrastructure and transporters [187], causing a temporary loss of CSF homeostasis. Further analyses of hypertensive and ischemic insults to the choroidal circulation should delineate repair mechanisms [125] that restore homeostasis of the CPe and ventricular volume.

### 5.3 Neuroendocrine factors

Peptidergically-regulated fluid transfer at the BCSFB and BBB [200,201] helps to set the volume and composition of CSF and brain ISF, respectively. Moreover the fine tuning of fluid exchange at the ependymal interface, mediated by neuropeptides [202] and growth factors [125], effects fluid balance between the ventricles and interstitium. When these coordinated homeostatic mechanisms at the transport interfaces are upset, the ventricles can either expand or contract.

At the blood-CSF interface in CP, there is a complex interplay among ANP, AVP and FGF2 and their apical membrane receptors to control ion and water secretion into the ventricles. This trio of peptides down-regulates CSF formation via neuroendocrine effects on CPe [2,6,17,81-87]. ANP, AVP and FGF2 levels in the CP-CSF system are mainly under central control [6,98,203-208] and thus largely independent of plasma and peripheral effects. When the CNS is perturbed biochemically or physically, these peptidergic systems are activated to restore normal hydration and osmolality. By these mechanisms, extracellular as well as intracellular fluid volumes in CNS are stabilized [209].

Ventricle size is affected by peptide regulation of CSF output from CP. FGF2 has many physiological roles, but a newly appreciated one is fluid regulation. Exogenously administered FGF2 both *in vivo *and *in vitro *reduces CSF formation [178,179]. This FGF2-induced suppression of fluid generation is evidently promoted by the choroidal release of the CSF inhibitor, AVP [210]. FGF2 and AVP expression in the CPe, like that of the hypothalamus-pituitary axis, is sensitive to the water balance of the organism [208,211]. It is intriguing that in the growing SHR the continually declining FGF2 level in CPe (and perhaps a resultant diminishing inhibitory tone on CSF production) is temporally associated with progressively increasing ventricle size [195]. Collective evidence implicates choroidal FGF2 in ventricular volume regulation.

ANP is also regarded as a volume-regulating peptide [212]. Several investigators report that choroidal ANP receptors in the ventriculomegalic SHR rat are down regulated [103,213-216]. Therefore, the reduced ANP tone lowers the generation of the CSF-inhibiting second messenger, cGMP, in the CPe of SHR [103]. A downloader of extracellular fluid volume in peripheral systems, ANP may have a similar function in the CNS. ANP receptor impoverishment in the CP of SHR rats [103,213-216] may cause less inhibition of CSF production and the related ability to maintain normal ventricular volume. Accordingly, the observed increase in CSF formation in the SHR [196], possibly due to attenuated natriuretic modulation, would be consistent with ventriculomegaly. The question is begged as to whether a genetic alteration in the ANP (or related neuroendocrine) systems in SHR rats precludes CSF volume down-regulation.

To construct a global model of CSF-ISF volume homeostasis, more information is needed on how ANP, AVP and FGF2 work in concert to regulate fluid transfer across CP and BBB [200,201,209]. AVP also interacts with angiotensin II in the CP-CSF system to decrease fluid production [217]. Neuropeptidergic modulation at fluid reabsorption sites in the CNS importantly awaits elucidation. Gaining insights on peptidergic regulation of CSF flow in various regions may make feasible the use of natural ligands or their analogs and antagonists [17] to therapeutically correct aberrant ventricle size.

## 6 CSF turnover rate

The brain's ability to turn over or renew CSF is crucially important in maintaining metabolic balance in the CNS. This is because the brain lacks lymphatic capillaries. It therefore relies heavily on continual CSF convection to clear macromolecules and catabolites from the neuronal environment. Such clearance deficiency, as in states of CSF stagnation when turnover rate is severely compromised, results in catastrophic changes in the composition of the interstitium, vessel walls and meninges. Consequently, neurons incur damage. Pharmacotherapeutic strategies to stabilize the aging CNS might well include ways to maintain CSF formation and prevent ventricular enlargement.

The CSF turnover rate is defined as the volume of CSF produced in 24 hours divided by the volume of the CSF space. In young adult rats, the total CSF volume is renewed about 11 times daily compared to 4 times/day in control humans (Table 4). Moreover, in both rodents and man, the rate of turnover of CSF declines with age and dementia progression (Table 4). Such dwindling of the CSF turnover rate is partly due to the declining ability of CP to form CSF in later stages of life [18,79].

**Table 4:** CSF dynamics and volume in aging and neurodegeneration

	Rat Aging*			**Human Disease^†^**		
	
	**3 mo**	**19 mo**	**30 mo**	**Normal**	**NPH**	**AD**
**CSF Formation Rate (ml/min)**	0.00121	0.00148	0.00065	0.40	0.25	0.20
**CSF Volume (space) (ml)**	0.156	0.196	0.308	150	300	250
**CSF Turnover Rate (volumes/day)**	11	10.8	3.0	4	1.2	1.2

* CSF formation rate and volume in rats were determined by indicator dilution during ventriculo-cisternal perfusion [79]. CSF turnover rate = formation rate/volume.

^† ^CSF formation rate and volume in patients were quantified by a modified Masserman technique [3]. NPH, normal pressure hydrocephalus; AD, Alzheimer's disease.

Volumes/day = the number of times each day that the entire CSF volume is renewed.

### 6.1 Adverse effects of ventriculomegaly

Another substantial factor in turnover rate is the volume of CSF. In young adults, there is a CSF space of 150–160 ml and a formation rate of about 0.4 ml/min [3]. The result is a normal turnover rate in man of about 4 volumes of CSF per 24 hours (Table 4). CSF volume or space however is augmented in senescence and dementia, often as an *ex vacuo *hydrocephalus secondary to atrophic loss of brain mass. Large ventricles inherently impair the CSF's ability to efficiently renew itself because turnover rate is inversely related to CSF volume (ventricles, cisterns and subarachnoid spaces). Accordingly, in aging, NPH and AD (Table 4), the CSF turnover rate can fall by 3–4-fold. Consequently as the ventricles enlarge in aging and disease [3], the purity of CSF is gradually lost due to less rapid turnover causing stagnation of extracellular fluid.

### 6.2 Attenuated CSF sink action

CSF disorders in both the young and old CNS often involve impoverished fluid turnover. CSF sink action to remove catabolites is a function of fluid formation and CSF volume relative to brain volume. In perinatal development when BCSFB transport capacity is less, the CP forms fluid at a slower rate per mass of tissue than in adults [33,106,107,110-113]. Moreover, in early life the ratio of CP-CSF volume to brain volume is relatively high [34]. These factors lessen the capability of immature brain to eliminate unneeded catabolites. The growing anabolic brain requires an optimally functioning CP-CSF to remove catabolites as well as provide micronutrients. Congenital hydrocephalus, with expanding ventricles, further reduces the already low degree of CSF sink action in the fetus [33,113]. Consequently, the low CSF turnover in infantile hydrocephalus makes the maturing CNS especially vulnerable to damage.

**Figure 8:**
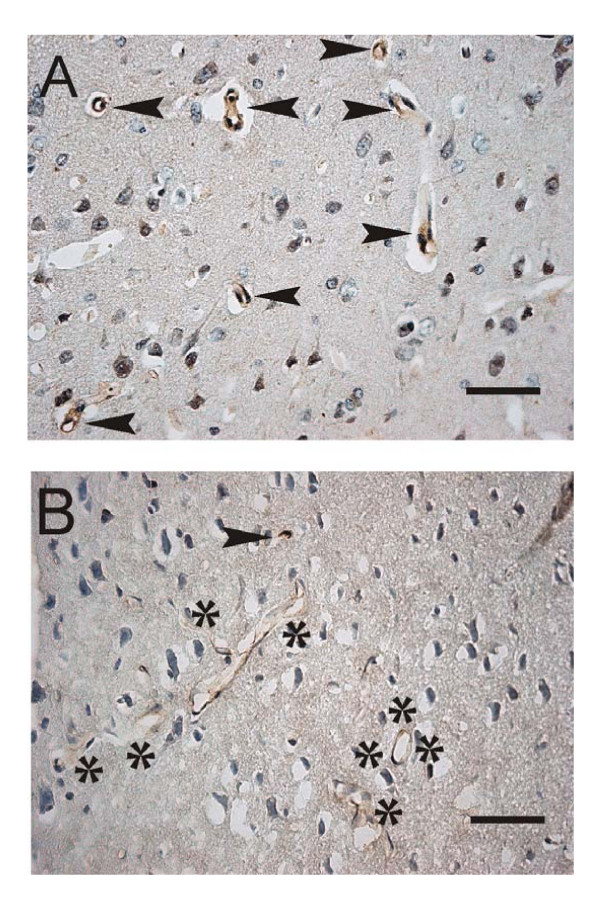
**Decreased expression of LRP-1 transporters in cortical vessels of rats with chronic hydrocephalus.** A. Immunostaining (bold arrows) of lipoprotein receptor-related protein-1 (LRP-1) in endothelial membranes of sectioned capillaries and arterioles in cerebral cortex of Sprague-Dawley rats (1-year control) B. Attenuated LRP-1 staining (negative vessels surrounded by asterisks) after 6 wk of hydrocephalus induced by kaolin injection into cisterna magna. Findings suggest reduced Aβ removal from cortex to blood in chronic hydrocephalus. Bar = 50 μm. Images reproduced with permission from *NeuroReport *[219].

In later life, CSF turnover rate is also markedly curtailed [2,3,9,18,19,79]. In senescence, NPH and AD, there is progressively lower CSF formation rate and expanded ventricular volume. The net result for all three states is diminished CSF turnover rate (Table 4). Such attenuated CSF sink action in aging, NPH and AD [3,19] alters CSF composition (see below) with deleterious consequences for brain. In aging, when dwindling fluid percolation and interstitial deposits hamper clearance of brain solutes, the curtailed flux of neurotransmitter metabolites (such as homovanillic acid) from interstitium to ventricles [218] likely reflects diminished bulk flow and diffusion between ISF and CSF. In a rat model that mimics NPH [219], beta amyloid peptide (Aβ) accumulates centrally as in NPH patients. In both cases Aβ clearance pathways are impaired (Fig. 8), by reduced transport of low-density lipophilic protein receptor related protein (LRP-1) at the BBB [219] and slower bulk flow from attenuated CSF dynamics [3,79]. Pharmacological intervention [11,12,15-17] in geriatric patients to maintain CSF formation and minimize ventricular dilation might prevent curtailed CSF turnover. A worthy goal is to provide trophic drugs or CP transplants [12,15] to minimize neurodegeneration by stabilizing CSF volume and content.

## 7 CSF composition

Historically, CSF compositional analyses have been used widely to monitor distortions in brain metabolism, evaluate disruptions of barrier transport and permeability functions, obtain pharmacokinetic parameters for drugs targeting the brain parenchyma, and identify biomarkers for aiding the diagnosis and prognosis of CNS diseases. Thus the CSF contains valuable biochemical and cellular information that can be advantageous for more effective clinical management of brain complications. An appreciation of altered CSF composition, especially in relation to corresponding changes in plasma, is particularly useful in interpreting scientific and neurological data.

### 7.1 Kidney-like action of CP-CSF system

The brain vitally depends upon the stability of CSF composition [220], similar to the way the body is dependent on plasma chemistry that is stabilized within tolerable limits. Acting like a kidney inside the CNS [34], the CP has a major role in homeostatically adjusting the brain extracellular fluid [1,18,27]. CSF constituents are exquisitely regulated by a complex array of transporters in the CP epithelium [1,18,46,68,220,221]. Substantial deviations in CSF concentration of ions and peptides require restoration to normality [17]. When CSF homeostasis fails, neuronal function becomes vulnerable [18,19,222]. A major translational challenge is to correct CSF solute distortions in aging, NPH and AD when both CP and BBB transport capacities are diminished [3,9,18,79,219,223]. Stabilizing the CSF composition is predicated largely on pharmacological manipulation of the barrier transporters and on restoration of a breached BCSFB or BBB [223-225]. To help manage certain CSF disorders, aberrant concentrations of proteins [226] and catabolites [174] in CSF need to be profiled and categorized for various stages of NPH and AD.

### 7.2 Altered CSF biochemistry in aging and disease

Biochemical analysis of the CSF has immense potential for diagnostics and prognostics [227]. In disease states the CSF composition is transformed by invading immune cells, plasma cytokines and pathogens [5]; as well as by modified active secretions from CP. Therefore the CSF is clinically useful to evaluate: i) the nutritional and trophic status of the brain in patients with hydrocephalus or dementia [228]; ii) the purifying capacity of central anion and peptide reabsorptive systems in aging and AD [229], iii) the therapeutic or cytotoxic concentration of certain CSF-borne agents targeted to neurons or meninges [230], and iv) the presence of abnormal solutes [231], or normal molecules in atypical concentrations. Solute concentration profiles can define certain disease states and be used to evaluate the benefit of therapeutic intervention.

**Figure 9:**
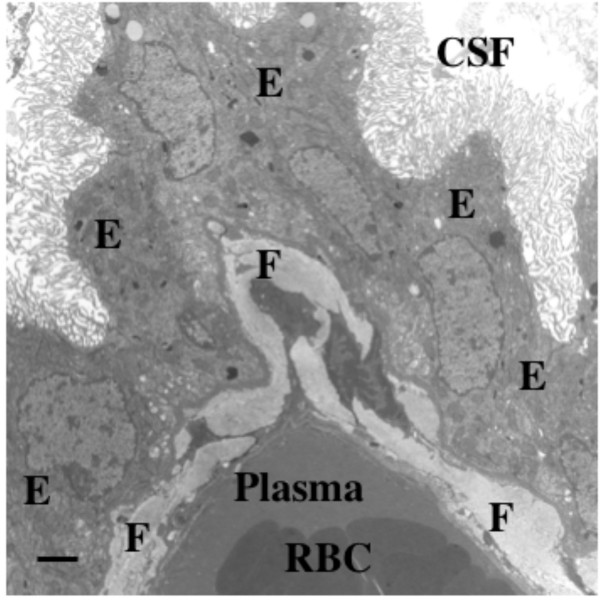
**Fibrosis in senescent rat choroid plexus.** Aging takes a toll on choroid plexus, functionally and structurally. This electron micrograph depicts massive collagen deposits in CP interstitium, i.e., between the vascular core and outer epithelial (E) ring. Fibrotic (F) bands in a 36-mo-old Brown-Norway/Fischer rat are 40–50 times thicker than corresponding collagenous layers in young adults. Excessive fibrosis likely impedes nutrient flow from plasma to ventricles and reabsorption of Aβ peptide fragments from CSF. Fibrosis in aging and AD also occurs in the arachnoid [240]. Consequently, fibrosis interferes with CSF dynamics via multiple effects. Appreciation is extended to P. McMillan for electron microscopy. Scale bar = 2 μm.

To sustain the brain, the CSF provides a full complement of vitamins, peptides, nucleosides and growth factors [1,2,10,27,222,232]. Adequate nutritional/trophic support of neurons via CSF is compromised in senescence [79]. This may result from dietary deficiency coupled with reduced micronutrient delivery to brain by inefficient CP-CSF transport mechanisms [18,57,223]. Vitamins B and C are transported specifically across CP to CSF and adjacent neural tissue [222-234]. In aging, massive interstitial fibrosis of CP (Fig. 9) and oxidative damage to the epithelium [223] impair this essential transport route. Structural and biochemical disruptions may reduce not only CSF formation rates, but also the secretion of organic substances into CSF of AD subjects [228]. Overall then, CSF concentration data for the elderly should be interpreted in light of reduced bulk flow as well as altered metabolism [218].

Proteins in CSF can also become deficient in late life. Transthyretin (TTR) is extensively synthesized and secreted by CP into CSF. TTR is a useful CSF marker because it is manufactured mainly by CP [235] and only slightly if at all, by brain parenchyma [236]. One function of TTR is to bind Aβ, thus stabilizing it in a soluble form and preventing CNS peptide toxicity [223] that may occur when Aβ self-assembles into neurotoxic oligomers. CSF levels of TTR decrease in both aging and AD [237,238]. A CSF deficiency of TTR may predispose the brain interstitium to Aβ oligomer toxicity and plaque formation [223]. Strategies are needed to sustain TTR secretion by CP in the elderly, e.g., by nicotinic modulation [239]. Moreover the array of growth factors secreted by CP helps to repair injuries from forebrain ischemia or neurodegeneration [7,17,125,126]. Brain fitness requires a sustained choroidal secretion of a cocktail of peptides and proteins that effect a balanced metabolic homeostasis of the CSF and neuronal-glial networks.

In addition to receiving productive secretions from the CP, the CSF is also a repository for catabolites diffusing out of brain. Excessive metabolite efflux from ISF to ventricles can toxify the CSF. Toxic catabolites in CSF, generated by peroxidative phenomena, increase with age and neurodegeneration [174]. Glycated proteins in CSF are elevated in AD [231]. The CP tissue that protects and nourishes the CSF-brain nexus, suffers from oxidative injury and disrupted metabolism in advanced age and AD [18,223,240,241]. A vicious cycle ensues as toxic neuronal products (e.g., Aβ) enter CSF from brain and inflict further harm to the CP already debilitated by aging [57,79]. The steady build up of Aβ in CP epithelium may disturb leptin transport from blood to CSF [242]. Such altered transport at the BCSFB could perturb neuroendocrine balance by interfering with hormone signal transfer to hypothalamus via the CSF.

### 7.3 Importance of clearance transport

Maintaining ISF and CSF compositional purity is of prime importance and depends upon efficient clearance mechanisms. Because the CNS lacks lymphatic capillaries, the CSF-mediated removal of metabolites and toxins is essential for efficient neuronal function. The CP-CSF system, proximally and distally, plays a major role in removing harmful substances. This occurs three ways. First, reabsorptive transporters in the apical membrane of CP epithelium actively remove organic anions and peptides from ventricular CSF [1,17,243]. This choroidal clearance complements active reabsorption of unneeded metabolites from ISF into cerebral capillaries [229]; for a given catabolite, the proportional clearance across BBB vs. BCSFB [244] should be determined in both health and particular diseases. Secondly, the continually-elaborated nascent CSF streams through the brain interior acting as a sink for metabolites diffusing down concentration gradients into the ventricles [1-3,33]. These substances pass through the ventriculo-subarachnoid system by bulk flow to distal drainage sites [1,18]. A third aspect of toxin clearance is the downstream extrusion of metabolites via outward CSF flow at the arachnoidal-lymphatic-venous interfaces [1,7,245]. In healthy CSF these three disposal mechanisms work in concert to effect fluid homeostasis. Therefore, in chronic hydrocephalus (NPH) with decreased CSF turnover [3,19], distorted CSF chemistry may be rectified by enhancing CSF formation, flow and drainage. Metabolite clearance defects and toxicity must be considered along with known ischemic insults in NPH to obtain a complete picture of chronic hydrocephalus.

Comprehensive analysis of CSF biochemistry helps to manage NPH, AD and other complications of senescence. Several animal models demonstrate inhibitory effects of neuropeptides and growth factors on CSF formation and reabsorption [178,246]. A logical extension is to analyze systematically the biochemistry of human CSF at stages of chronic hydrocephalus with and without neurodegeneration. CSF profiling of protein, peptides and organic metabolites (e.g., sphingomyelin derivatives) in aging patients will likely provide insight on how the failing barriers and brain metabolism are linked to deficient CSF dynamics. Elevated levels of ventricular CSF albumin, neuropeptide Y, vasoactive intestinal peptide and sulfatide (an ischemia marker for subcortical arteriosclerotic encephalopathy) have negative implications for surgical outcome by shunting [247,248]. Systematic analyses of CSF constituents in progressive iNPH and aqueductal stenosis [247] will likely improve predictive power for shunting benefits.

### 7.4 Therapeutic manipulation of composition

Pharmacological manipulation of CSF and brain ISF composition may prove feasible. One goal is to improve CSF flow and content through actions of drugs delivered across the BCSFB into the brain interior [16,17]. Reparative agents are needed in periventricular regions (e.g., the subventricular zone, hippocampus and white matter) damaged by elevated CSFP [91], ischemia [7,126,186,187] and distorted CSF chemistry. Another compelling need is to develop CSF-cleansing procedures for geriatric patients in whom brain fluid turnover is seriously compromised. This would be analogous to renal dialysis in patients with kidney failure. Novel technology for CSF dialysis or low-flow shunting, in particular subgroups of patients with dementia, may improve cognition. Therefore, the use of new drugs [16] and biotechnological devices [15] deserves consideration, to slow down degeneration or promote healing of NPH/AD. CPs and ventricles [16,17] show promise as regulatory sites to attain desired drug concentrations and optimize CSF composition [249].

## 8 CSF recycling in relation to ISF dynamics

Functional interactions between CSF and ISF are plentiful. CSF and ISF move by bulk flow, the former through large cavities and the latter finely through narrow winding pathways in the neuropil and grossly along myelinated fiber tracts. Fluid is convected into and out of the brain parenchyma, depending upon prevailing local hydrostatic pressure gradients. Knowledge of bulk flow pathways is important for: the delivery of micronutrients and peptides to neurons and glia, the conveyance of hormone signals to target cells, the delivery of drugs to receptors, and the removal of catabolic products from CNS. Pathological sequelae ensue when ISF and CSF do not freely percolate. CSF movement via interstitial pathways requires further analysis due to disease implications.

### 8.1 CSF exchange with brain interstitium

CSF and ISF dynamically exchange water and solutes across the ependymal (interior) and pia-glial (exterior) surfaces of brain [1,27]. Brain ISF has distinctive circulatory features [22]. Continual ISF turnover, even at a relatively slow rate [250,251], maintains an optimal microenvironment for neurons. In aging there is less efficient active transport at the brain capillary endothelium and choroidal epithelium. Consequently, the slower turnover of ISF and CSF causes CNS accumulation of potentially injurious organic acids and peptides. Neurons are harmed in senescence when metabolites accumulate interstitially [9].

### 8.2 Components of ISF movement in brain

Metabolic waste is discarded through ISF and CSF clearance routes. Brain ISF conducts metabolites away from neurons and glia to excretory sites. ISF has multiple input and output components that keep the fluid mixed and delivered to excretory loci. On the input side of ISF volume and flow is a CSF-like fluid likely secreted by the astroglial-endothelial complex in microvessels [252]. The putative BBB fluid production is much less than by CP. Another input to ISF is water generated by parenchymal metabolism [252]. A third input to ISF is a fraction of the SAS fluid (originally derived from the CP-ventricular nexus) that enters cortex via the Virchow-Robin perivascular spaces [253-255], propelled in part by the pumping action of arteries and arterioles. As such, this recycled CSF likely drains back into the ventricles.

ISF output has at least two components. First, quantitatively substantial reabsorptive fluxes of solutes (e.g., Aβ peptides) occur via LRP-1across endothelial cells into vascular lumina [229]. As LRP-1 expression at the BBB wanes, as with age, the CP LRP-1 transporter may need to cover this deficit in clearance in brain capillaries by reabsorbing proportionally more Aβ at the BCSFB. Another class of solutes reabsorbed across BBB is organic acids/anions, actively removed from brain to blood by the organic anion transport system (OATS) [256]. Secondly, there is bulk flow and diffusion of substances from ISF to large-cavity CSF [22] that are ultimately reabsorbed at arachnoidal sites. Studies are needed to determine how the proportion of catabolite clearance, at BBB vs. BCSFB and bulk flow, changes with progression of brain pathology. Fluid percolation through brain interstices [251] is substantially slower than CSF circulation through ventriculo-subarachnoid spaces. ISF bulk flow with its entrained catabolites awaits comprehensive characterization, particularly in regard to ISF interaction with the water dynamics of CSF.

### 8.3 Compromised ISF/CSF dynamics and amyloid retention

Volume transmission of ISF and CSF is substantially attenuated in aging and dementia [3,9]. Brain catabolites build up when fluid turnover rates fall by > 50% [19]. An example is Aβ40, generated by enzymatic cleavage of amyloid precursor protein [229]. Due to deteriorating fluid dynamics, Aβ accumulates in ISF and perivascular spaces during aging, NPH and AD. Our working model is that central Aβ clearance is compromised by interacting factors in aging. By nine months in the mouse there is reduced efflux transport of radiolabeled Aβ1-40 [229], perhaps due to decreased LRP-1 transport at the abluminal interface of the BBB. LRP-1 loss would result in slower removal of Aβ from ISF [229], causing Aβ42 to accumulate in cortical microvessels [257]. Moreover, attenuated CSF formation reduces ventricular sink action [33] that draws (by concentration gradients directed into CSF) Aβ fragments and other peptides from ISF. Another unfavorable outcome in senescence is Aβ42 accumulation in CP epithelium, ependyma and arachnoidal cells [257]. These CSF-bordering interfaces control fluid movement among CNS compartments [1]. When burdened with toxic Aβ, the choroido-meningeal tissues are probably less effective homeostatically in stabilizing brain ISF against plaque formation.

Cumulative evidence for dwindling transport and fluid dynamics in aging, NPH, and AD, [3,19] points to common vulnerabilities in the degenerating BCSFB [18] and BBB [224]. Hydrocephalus induced in old rats by kaolin injection causes Aβ retention in many CNS regions [219]. A unified model for brain proteinopathies should relate peptide/protein distribution kinetics to fluid-turnover capacities. ISF functional integrity relies upon an efficient CP-CSF system and healthy cerebral microvessels. Following a late-life breakdown of transporters, the brain interstitium accumulates catabolite debris, including peptide fragments. This predisposes to concentration-dependent Aβ self-assembly into oligomers, fibril formation and plaque deposition. Information is needed on how Aβ retention alters CBF, arteriolar actin [258], hydraulic conductivity of ISF, tortuosity of extracellular channels and viscoelasticity of the matrix. These factors impact the interactive ISF-ventricular fluid dynamics and hence the delivery of catabolites to excretory pathways.

## 9 CSF reabsorption

CSF clearance into lymph and blood involves diverse anatomical sites and physiological mechanisms. Disrupted CSF reabsorption is a common cause of hydrocephalus. The eventual resolution of clinical hydrocephalus depends upon knowledge of outflow sites and their regulation. When CSF is retained in the CNS, compensatory mechanisms stabilize ICP [97]. In addition to cranial nerve outflow adjustments, there are spinal nerve arachnoidal pathways that facilitate fluid egress following CSF retention in the spinal SAS. Moreover, when CNS is fluid overloaded, the brain capillaries may use up regulated AQP channels to absorb ISF under high pressure. Improved understanding of fluid reabsorption at multiple sites will lead to better management of CSF and ISF congestion.

### 9.1 Arachnoidal outflow resistance

CSF Ro has been the key hydrodynamic parameter to evaluate reabsorptive phenomena. Methodologically, CSF infusions accompanied by CSFP measurements have generated ample Ro data for humans and animals [259]. In rodents, Ro is elevated in congenital hydrocephalus [115,260] and NPH-like states [178]. CSF Ro increases two- to three-fold in patients with NPH [261]. The elegant work of Jones and colleagues in rats [105,262] demonstrated that in normal CSF development, the Ro to CSF reabsorption across the transverse sinus decreases between 10 and 20 days postnatal, i.e., when upstream CSF secretion increases [33,113]. However, in hydrocephalic H-Tx rats, Ro (from lateral ventricle) increases after birth due to aqueduct occlusion [115] but does not decrease in later development as in non-hydrocephalic controls. Recently, Eklund and colleagues [259] reviewed important applications of Ro data derived from CSF infusions.

### 9.2 Arachnoid villi vs. olfactory drainage routes

**Figure 10:**
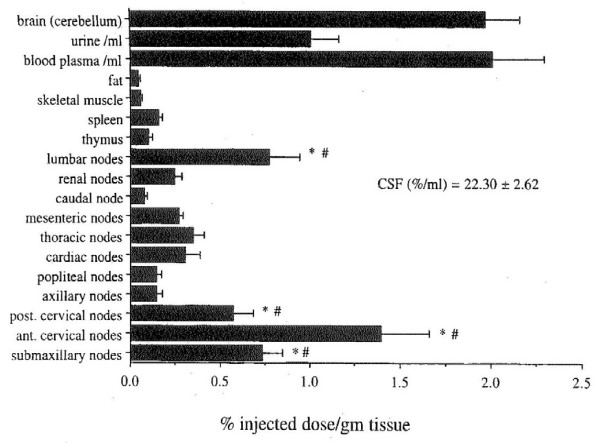
**CSF reabsorption routes in the adult rat as revealed by recovered 125-labeled human serum albumin (HSA) in lymph nodes:** Anesthetized male adult Wistar rats (n = 8) were killed 6 hr after ^125^I-HSA injection into lateral ventricle. HSA uptakes were averaged for bilateral and multiple nodes. Symbols indicate differences (*P *< 0.05) in test node activity relative to spleen (*) or popliteal (#) lymph nodes. Head and lymph node activities greater than spleen indicate tracer passage from CSF directly to submaxillary or cervical nodes. Lumbar node activity was above the spleen and popliteal nodes (not expected for direct CSF drainage). Reproduced with permission from M. Johnston and the *American Journal of Physiology *[268].

There has been lively debate regarding the main site of CSF reabsorption. The past decade witnessed a gradual paradigm shift on the preponderant site of CSF drainage in several animal models. Accordingly, drainage/reabsorption of CSF via the olfactory [263] and optic nerves [264], cribriform plate, nasal submucosa and cervical lymphatics is now regarded as the primary bulk flow passageway for extruding subarachnoid CSF from the brain of rats, pigs and sheep [265-272]; and even in a non-human primate [269]. Microfil injection studies by Johnston and colleagues provide substantial evidence that CSF flow into nasal lymph is the major route of CSF egress in several species [266]. The prevailing view is that lymphatic vessels external to the cranium receive a large volume of CSF in many mammals (Fig. 10); however, humans have yet to be evaluated *in vivo*. This new emphasis on drainage via the olfactory route [265-272], rather than by arachnoid villi, culminates a century-long notion that CSF and lymph have a functionally intimate association.

Until recently, CSF drainage models have emphasized a substantial role for the arachnoid villi embedded in the dura mater of the superior sagittal sinus. Each arachnoid villus was thought to have a one-way (CSF outward) valve-like mechanism that opened in response to a positive hydrostatic pressure gradient between CSF and dural venous blood [252]. It now appears that in most mammals the arachnoid villi normally are not the locus of most CSF reabsorption, but under conditions of elevated CSFP may mediate an increasing amount of CSF clearance [267]. In man, the physiological role of arachnoid villi in the setting of healthy CSF dynamics awaits characterization, as do the precise mechanisms of CSF flow along cranial nerves to the eyes and nose. Large Pacchionian granules in CSF of humans [252], compared to diminutive arachnoid villi in rodents, suggest the former may play a relatively greater role in CSF reabsorption in man.

Johnston and colleagues have implicated the cribriform plate above the nose as a key extracranial site in CSF outflow. Assessments of proportional flow through cribriform and non-cribriform routes indicate that at low ICP the CSF absorption occurs mainly across the plate [269]. Thus an experimentally sealed cribriform plate is useful for evaluating CSF hydrodynamic parameters under conditions of blocked CSF flow into the nasal submucosa and cervical lymphatics. An obstructed cribriform plate substantially reduces CSF clearance, increases ICP [270] and impairs regulatory systems that compensate for CSF volume infusions [269]. At least in sheep, the abovementioned global CSF parameters for late gestation resemble those in adults [271], including the relative non-involvement of arachnoid villi vs. the more functional olfactory drainage pathways [272]. Brinker et al. [273], using magnification radiography during infusion of adult rats, earlier demonstrated a significant rapid drainage of CSF via the olfactory, optic and cranial nerves (VII and VIII) into cervical lymph.

**Figure 11:**
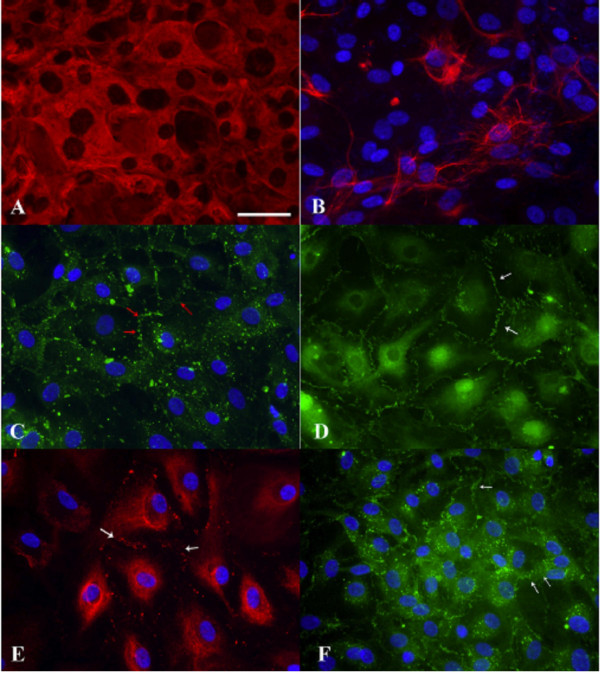
**Immunostaining of cytoskeletal and junctional proteins in cultured human arachnoid granulation epithelial cells.** A. Expression of intermediate filament protein vimentin (Cy3 conjugated anti-vimentin antibody). B. Expression of cytokeratin (FITC conjugate to broad spectrum anti-cytokeratin antibody). C. Expression of connexin43 (FITC conjugate); punctate distribution at borders (red arrows) points to gap junctions in cell culture. D. ZO-1 (tight junction) staining (FITC conjugate) at cell-cell borders (white arrow) demarcates overlapping filapodia (short linear structures in parallel). E. Expression of desmoplakin (with Alexa Fluor 555 conjugated secondary antibody) reveals desmosomes along adjacent cell borders (white arrows). F. Expression of E-cadherin, an epithelial-specific cell adhesion molecule, at periphery of cells (white arrows) in a pattern similar to that of connexin43 and ZO-1. All images have same scale (bar = 50 μm). Reproduced with permission of Holman *et al *[277].

Reabsorbed CSF ultimately drains into venous blood. Therefore, venous occlusion experiments provide clues on the relative contribution of particular CSF-venous interfaces to CSF reabsorption. Hydrocephalus (CSF retention) has been associated with impaired extracranial venous obstruction [274]. Collectively, such observations point to olfactory/cervical lymphatic drainage, rather than arachnoid villi, as the primary mechanism of normal CSF drainage [245]. Moreover, arachnoid villi and granulations may serve as an ancillary CSF drainage route (by opening one-way valves) when ICP rises substantially. This fits earlier observations of hydrostatic pressure gradient-dependent bulk flow across arachnoid villi [252] and the formation of fluid-filled vesicles in arachnoid epithelium under elevated pressure. Pore-like openings were observed in a thin neurothelial layer along the optic nerve extending into the lymphatics [275]. Promising investigations of human arachnoidal granulations *in vitro *(Fig. 11), utilizing chamber and cell culture preparations such as those devised by Grzybowski *et al*. [276,277], should elucidate fluid transport across hydrostatic pressure-sensitive pores. Answers are needed for key questions: How do arachnoid cell pore-like structures respond biophysically (ultrastructurally) to elevated CSF and/or venous pressure? Also how are the biochemical secretions of the arachnoid membrane altered by intracranial benign hypertension or hydrocephalus? Biophysical and biochemical data for cranial as well as spinal CSF reabsorption await integration.

### 9.3 Fluid reabsorption along spinal nerves

CSF reabsorption also occurs along spinal nerves. Radionuclides injected into the CSF in a cisternography study of human reabsorption revealed a 20% reduction of radioactivity in the spinal subarachnoid space during one hour of testing [278]. This spinal CSF clearance was enhanced in physically active (vs. resting) individuals. When hydrocephalus was induced in adult rats by cisternal kaolin injection, resulting in blocked cranial absorption of CSF, there was compensatory fluid clearance along lumbo-sacral rootlets [279]. In another kaolin study producing hydrocephalus/syringomyelia in rats, high-molecular weight ferritin traced the bulk CSF passage from the central canal, across ruptured ependyma and dorsal columns, and then along spinal nerves into extradural lymphatic vessels [280]. Such compensatory spinal CSF outflow might be particularly significant in upright humans [280].

### 9.4 Reabsorption across capillary aquaporin channels

Hydrocephalus impacts and is impacted by CSF egress. When increased Ro causes central fluid overloading, there may be alternate non-arachnoidal mechanisms activated by augmented ICP to prevent further build up of CSF and ISF. Water channel AQPs at the blood-CSF [78] and blood-brain interfaces are significant control points to regulate fluid movements. AQPs 1 and 4, respectively, are expressed in CP and brain microvessels. When cranial nerve outlets, arachnoid villi and spinal nerve reabsorptive mechanisms are overwhelmed by extra fluid, compensatory changes in AQP expression may participate in ICP adjustments. Thus, the elevated CSFP attending hydrocephalus (in both kaolin-induced and the H-Tx genetic anomaly), leads to augmented expression of AQP4 [281-283]. A key question is whether up regulated AQP4 in hydrocephalic brain enhances ISF reabsorption into capillaries. On the other hand, when AQP1 at the BCSFB is down regulated, there is less fluid input to the CNS because CSF formation is reduced and ICP consequently lowered [77,78]. Altered AQP expression at the barriers implies that water fluxes via channels that contact ISF and CSF can be pharmacologically regulated to remediate brain fluid imbalance secondary to faulty reabsorption.

## 10 Developing translationally effective models for restoring CSF balance

Restoration of fluid balance perturbed in congenital and adult chronic hydrocephalus remains an enormous challenge. Translational progress is difficult because CSF is complexly interactive with many fluid compartments and interfaces (Fig. 1). In elucidating CSF dynamics, it is imperative to analyze not only the CP-CSF-arachnoid nexus [284] but also the brain capillary-ISF-ependymal nexus. A thorough understanding of fluid transfer from brain to CSF to lymph, or in the reverse direction during elevated-pressure hydrocephalus (from CSF to ISF to cerebral capillary blood), should expedite therapy with drugs or fluid-drainage hardware.

**Figure 12:**
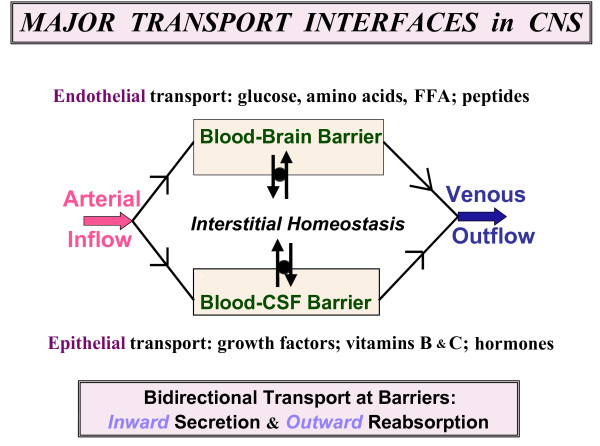
**Concerted transport at blood-brain and blood-CSF barriers:** Cerebral capillaries and choroid plexuses transport different materials [16, 17, 27, 34]. Although there is transport overlap, the BBB largely supplies brain with glucose, amino acids, free fatty acids (FFA) and peptides, whereas BCSFB furnishes the CSF-brain nexus with vitamins, growth factors and proteins such as transthyretin. CNS fluid balance results from regulated water transport across AQP4 at BBB and AQP1 in BCSFB. The reabsorptive transporters LRP-1 and P-glycoprotein (Pgp) are expressed differentially in BBB [225, 229] and BCSFB [291]. Both interfaces have to be analyzed to characterize phenomena such as Aβ deposition in interstitium. To understand brain fluid composition and barrier interaction pathophysiologically, it is advantageous to analyze BBB and BCSFB concurrently.

The CPs are more than an appendage of the BBB [1]. Rather, the composite BCSFB is a major interface that, when functioning in parallel with the cerebral capillary network (Fig. 12), effects a stable extracellular environment for neurons at optimal volume, composition and pressure. Having a renal-like function to stabilize fluid in the CNS [34], the CP protects the brain just as the kidney safeguards peripheral organs by stabilizing the plasma constituents. To maintain CNS stability, is there physiological cross talk or signaling between BBB and BCSFB? Probably so, but multi-compartmental analyses are necessary to answer this question.

An altered BCSFB can affect the BBB, and *vice versa*. The actions and responses at these primary transport interfaces are addressed with *in situ *and *in vivo *models allowing concurrent analyses of barriers not possible with *in vitro *and cell culture preparations. One approach is multi-compartmental analysis [112] or simultaneous microdialysis [285] of brain (parenchymal probe) and CSF (cisternal microprobe), following systemic administration of radiotracers [286]. Another tack is regional immunohistochemical analyses of peptide, channel or transporter expression in CP, brain capillaries, ependyma, neurons and arachnoid [219]. Systemic analysis of solute and water transfer enlightens the magnitude and vector of fluid shifts. Furthermore, Western blot, ELISA and PCR data from isolated CP and brain microvessels furnish complementary data [257] to characterize *in vivo *transport mechanisms.

A fascinating advance for CSF modeling is that the BCSFB and BBB can be involved in opposite responses. This happens when the CNS adapts to altered fluxes of water and peptides (e.g., resulting in Aβ retention) in diseases that physically impair distribution pathways. Table 5 shows an overview of the compensatory adjustments for aging, NPH and AD in regard to water and peptide translocation at blood-CNS interfaces. First, in regard to water movement through channels (Table 5A), there is normally net diffusion of water from blood to brain. In high-pressure hydrocephalus, however, there may be net reabsorption of fluid from ISF to capillary blood via up-regulated AQP4 channels. However, such a reversal phenomenon in the BBB as well as down-regulated AQP1 and CSF formation in hydrocephalus, need to be demonstrated functionally. Moreover, Aβ clearance from brain ISF to blood via capillary LRP-1 is evidently decreased in aging, NPH and AD, but may be increased or maintained in the reabsorptive CSF-CP nexus where LRP-1 is up-regulated or sustained in these three states (Table 5B). This postulate awaits proof from kinetic experiments. Still, mounting evidence indicates that the BCSFB and BBB are complexly interactive physiologically and in pathologic states. By integrating data for CP, ependyma, arachnoid and BBB vasculopathy, insight will be gained on how CSF-ISF dynamics impacts brain integrity [287].

**Table 5:** Differential expression of water channels and peptide transporters at the BCSFB and BBB: Implications for CNS fluid and peptide homeostasis

**A**	
**Aquaporin 1 (Blood-CSF Interface)**	**Aquaporin 4 (Blood-Brain Interface)**

AQP1 is present at the CSF-facing pole of choroid plexus epithelium [70 – 73, 77, 78], and in the CSF-brain ependymal lining [281].	AQP4 is located in astrocytic foot processes at the BBB. It is associated with fluid transfer across cerebral microvessels.
Reduced expression in choroid plexus is associated with slower rates of CSF secretion and thus decreases ICP [77, 78].	Elevated expression of AQP4 in the BBB occurs in chronic hydrocephalus [281 – 283]; may be associated with fluid reabsorption.
Attenuated expression in aging [57] and in Alzheimer's disease is accompanied by slower fluid turnover rate [79].	Diminished expression at BBB leads to a reduction in brain edema formation in some animal models.

**B**	

**LRP-1 **(Low density lipophilic receptor associated protein)	**RAGE **(Receptor for advanced glycation end products)

Expressed in choroidal epithelium and capillary endothelium [229, 257].	Expressed in choroidal epithelium and brain capillary endothelium [257].
Removes Aβ peptide from CSF and brain ISF [229, 243] for excretion via blood.	Transports Aβ from blood into brain ISF where the retained peptide may predispose to Aβ plaque formation in the interstitium.
Sustained or increased expression in choroid plexus during aging, NPH and AD.	Enhanced expression of RAGE at BBB in aging, NPH [219] and AD [225] may destabilize BBB and precipitate plaque.
Decreased expression of LRP-1 at the BBB in aging, NPH and AD likely interferes with Aβ removal [219, 225, 229].	Expression of RAGE is generally opposite to that of LRP-1 at the barriers and in neurons [225].

## 11 Conclusion

CSF integrates a multiplicity of functions for the CNS. From fetal life through adulthood, and extending into terminal stages, CP-CSF actively engages in building, maintaining and repairing the brain [288]. Efficient CSF homeostatic mechanisms are vital to neuronal networks. CSF dys-homeostasis in aging and illness, however, can compromise motor functions and cognition. Stabilizing or restoring BCSFB and BBB functions in senescence and disease challenges the next generation of investigators. Clinical translation of fluid homeostasis concepts is contingent upon expanded knowledge of the multiple transport interfaces that contact CSF.

## List of abbreviations

aAMP, arterial pulse amplitude; Aβ, beta-amyloid protein or peptide; AD, Alzheimer's disease; AE2, anion exchanger, e.g., Cl^-^-HCO_3_^- ^antiporter; ANP, atrial natriuretic peptide; AQP, aquaporin; AVP, arginine vasopressin; BBB, blood-brain barrier; BCSFB, blood-cerebrospinal fluid barrier (choroid plexus); c.a., carbonic anhydrase; CBF, cerebral blood flow; CBV, cerebral blood volume; cGMP, cyclic guanylyl monophosphate; CNS, central nervous system; CP, choroid plexus; CPe, choroid plexus epithelium; CSF, cerebrospinal fluid; CSFP, cerebrospinal fluid pressure; ELISA, enzyme-linked immunosorbent assay; FGF2, basic fibroblast growth factor; H-Tx, hydrocephalic rat model with a genetic origin and obstruction of the cerebral aqueduct; ICP, intracranial pressure; ISF, interstitial fluid; LRP-1, low-density lipophilic protein receptor related protein (Aβ transporter); MRI, magnetic resonance imaging; NHE1 and NHE3, sodium-hydrogen exchangers; NKCC1, Na-K-Cl cotransporter (secretory form); NPH, normal pressure hydrocephalus; PC-bSSFP, phase contrast balanced steady-state free precession; Pgp, P-glycoprotein transporter; RAGE, receptor for advanced glycation end products (Aβ transporter); RF, Reissner's fiber; Ro, resistance to CSF outflow; RPPC, relative pulse pressure coefficient; SAS, subarachnoid space; SCO, subcommissural organ; SHR, spontaneously hypertensive rat; SLC4, member of the bicarbonate transporter family; TTR, transthyretin.

## Competing interests

The authors declare that they have no competing interests.

## Authors' contributions

CEJ had the main responsibility for organizing and writing the manuscript. JAD aided in developing the material on clinical aspects of pediatric and adult hydrocephalus. PMK and TB contributed perspective on CSF pressure, kaolin hydrocephalus and CSF reabsorption. EGS helped to interpret pathophysiological findings on Alzheimer's disease. GDS provided insight on human and animal BBB transporter expression and CSF dynamics in aging, NPH and dementias; and had a major role in editing. All authors have read and approved the final manuscript.
